# Development of a Sealed Rechargeable Li–SO_2_ Battery

**DOI:** 10.1002/advs.202411598

**Published:** 2024-12-17

**Authors:** Gayea Hyun, Myeong Hwan Lee, Haodong Liu, Shen Wang, Zeyu Hui, Victoria Petrova, Ping Liu

**Affiliations:** ^1^ Aiiso Yufeng Li Family Department of Nanoengineering University of California San Diego La Jolla CA 92093 USA; ^2^ Program of Material Science University of California San Diego La Jolla CA 92093 USA; ^3^ Present address: Advanced Battery Research Center Korea Research Institute of Chemical Technology (KRICT) 141, Gajeongro Yuseong Daejeon 34114 Republic of Korea; ^4^ Present address: Department of Energy Science Sungkyunkwan University Suwon 16419 Republic of Korea

**Keywords:** electrolyte additives, Li–SO_2_ batteries, lithium protection, SO_2_ utilization, sulfur dioxide

## Abstract

Rechargeable Li–SO_2_ batteries offer low‐cost, high‐energy density benefits and can leverage manufacturing processes for the existing primary version at a commercial scale. However, they have so far only been demonstrated in an “open‐system” with continuous gas supply, preventing practical application. Here, the utilization and reversibility of SO_2_ along with the lithium stability are addressed, all essential for long‐life, high‐energy batteries. The study discovers that high SO_2_ utilization is achievable only from SO_2_ dissolved in electrolytes between the lithium anode and carbon cathode. This results from a unique osmosis phenomenon where SO_2_ consumption increases salt concentration, driving the influx of organic solvents rather than SO_2_ from outside the current path. This insight leads to configure a bobbin‐cell with all electrolytes between the electrodes, realizing nearly 70% of SO_2_ utilization, > 12x greater than in conventional coin cells. To improve reaction rate and SO_2_ reversibility, triphenylamine is employed to the electrolyte, creating an electron‐rich environment that alleviates the disproportionation of discharge products. Incorporating this additive into a bobbin‐cell with a lithium protective layer yields a cell with a projected energy density exceeding 183.2 Wh kg^−1^. The work highlights the potential of Li–SO_2_ batteries as affordable, sustainable energy storage options.

## Graphical abstract

Rechargeable Li–SO_2_ batteries provide affordable, high‐energy storage but are limited to open systems requiring continuous gas supply. This study introduces a sealed bobbin‐cell format, with all electrolytes placed between the electrodes, maximizing SO_2_ utilization. By incorporating an electrolyte additive and a lithium protective layer, the cell improves SO_2_ reversibility, demonstrating its potential as a more sustainable energy solution.

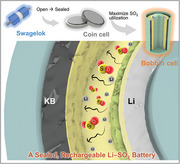

## 1 Introduction

Primary Li–SO_2_ battery is a mature commercial product featuring high‐energy density, wide operational temperature range, and exceptional shelf life. A rechargeable version would be highly desirable as well due to the abundance of raw materials, free of any resource contraints.^[^ ^1^, ^2^, ^3^, ^4^, ^5^ ^]^ SO_2_ can dissolve in organic electrolytes with high solubility which makes it feasible to construct these batteries for use at ambient pressure.^[^ ^6^, ^7^, ^8^ ^]^ It is particularly appealing to convert existing Li–SO_2_ primary batteries into rechargeable systems since the infrastructure for the storage, transport, and handling of SO_2_ is already in place.^[^ ^9^, ^10^ ^]^

In the presence of organic solvents, discharge of SO_2_ results in the formation of lithium dithionite (Li_2_S_2_O_4_), which is found to be reversible.^[^ ^11^, ^12^ ^]^ So far, research has focused on developing suitable electrolytes capable of stabilizing SO_2_^−^ intermediates prior to final Li_2_S_2_O_4_ formation by constructing strong solvation shells. This approach not only hinders irreversible electrolyte decomposition but also promotes the formation of reversible discharge products nuclei.^[^ ^13^, ^14^ ^]^ Carbonate‐ and ether‐based electrolytes, diglyme, and ionic liquids have been explored as electrolytes to improve solvation ability. In particular, highly solvating carbonate‐based electrolytes have recently demonstrated long cycle life.^[^ ^8^, ^15^, ^16^, ^17^ ^]^ Additionally, various carbonaceous materials, including reduced graphene oxide, Ketjen Black (KB), and activated carbon have been investigated as cathode materials to provide high surface area and pore volume for accommodating solid Li_2_S_2_O_4_ products.^[^ ^2^, ^18^, ^19^ ^]^ Manipulating electrode microstructure enhances catholyte mass transport, improving discharge capacity and reversibility. However, all these improvements have been implemented in an open system where SO_2_ is continuously supplied and lithium is in great excess.^[^ ^20^, ^21^ ^]^

The progress made in electrolyte and electrode architectures of secondary Li–SO_2_ batteries achieved in open systems have yet to translate to practical sealed batteries. In a sealed battery cell, the amount of SO_2_ is determined by its solubility in the organic electrolyte. Choosing the solvents that are highly miscible with SO_2_ is thus essential. On the other hand, the rate of the SO_2_ redox reaction and its cycling stability depend on designing the proper electrode microstructure and incorporating suitable catalysts,^[^ ^13^, ^14^, ^15^, ^22^ ^]^ just like in the open system. Finally, achieving stable long‐term cycling requires mitigation of lithium corrosion by SO_2_ through surface protection.^[^ ^23^, ^24^, ^25^, ^26^ ^]^

Here, we report electrolyte, electrode, and cell designs for sealed rechargeable Li–SO_2_ batteries with a focus on improving practical energy density and cycling stability. We study the mass transport dynamics in the sealed system and uncover the root cause that determines the supply of SO_2_ to the electrode. Based on these insights, we develop a bobbin‐cell where virtually all electrolyte is in between the electrodes. The design also features a protective layer to mitigate lithium corrosion and an electrolyte additive to increase the reversibility of the SO_2_ cathode and reaction kinetics. As a result, we achieve nearly 70% utilization of SO_2_ and a projected energy density of 183.2 Wh kg^−1^ for the cell. Our work lays the groundwork for developing a practical rechargeable Li–SO_2_ battery.

## 2 Results and Discussion

### 2.1 Investigation of Low SO_2_ Utilization and Pathways for Improvement

#### 2.1.1 SO_2_ Solubility and Evaluation in a Coin Cell

To prepare SO_2_‐containing electrolytes, we introduced SO_2_ gas into a vial sealed with a rubber stopper, containing 1 m lithium bis(trifluoromethylsulfonyl)imide (LiTFSI) in ethylene carbonate/dimethyl carbonate (EC/DMC) at a 1:1 volume ratio. We monitored the weight change over time while maintaining the pressure at the gas inlet at 15 psi. Figure S1a (Supporting Information) depicts the weight percentage of dissolved SO_2_ relative to the total mass of the electrolyte as a function of gas injection time. Within the first 5 min, 14.6 wt% of SO_2_ dissolves into the electrolyte, increasing to 25.5 wt% at 40 min after which no further increase in weight is seen. There is an increase in volume of 46.7% when reaching SO_2_ saturation; the density of the solution only experiences a marginal rise from 1.34 to 1.36 g ml^−1^ (Table S1, Supporting Information). Furthermore, there is a small decrease in the viscosity of the solution and a slight reduction in ionic conductivity (Figure S1b and Video S1, Supporting Information).

The redox of SO_2_ is first evaluated by cyclic voltammetry (CV) in a two‐electrode coin cell. Distinct oxidation and reduction peaks corresponding to the SO_2_ redox reaction at 3.65 and 2.81 V vs. Li/Li^+^ are observed (Figure S2, Supporting Information).^[^ ^27^, ^28^ ^]^ These are consistent with previous reports, indicating the chemical reversibility of the reaction. We next investigate the discharge capacity for electrolytes containing different concentrations of SO_2_. The 1.13 cm^2^ cathode is made of KB with a loading of 0.8–0.9 mg cm^−2^. The coin cells are assembled using 0.076 g (equivalent to ≈56.0 µL) of electrolyte. Previously work has shown that KB has a specific capacity of exceeding 6500 mAh g_KB_^−1^.^[^ ^1^, ^15^ ^]^ Thus, we do not expect the cathode will become capacity‐limiting during our tests. With an increase in SO_2_ concentration from 14.6 to 25.5 wt%, the specific capacity increases from 357.4 to 592.0 mAh g_KB_^−1^ at a current density of 0.2 mA cm^−2^ (Figure S3, Supporting Information). We calculate the utilization of SO_2_ assuming that all electron transfer reactions yield the ideal reversible discharge product, Li_2_S_2_O_4_ (Note S1, Supporting Information). Complete conversion of 0.019 g of SO_2_ (0.076 g of electrolyte at 25.5 wt%) corresponds to a capacity of 8.11 mAh. However, an actual capacity of 0.43 mAh is obtained, reflecting a utilization of 5.3%. The low SO_2_ utilization along with the substantial influence of its concentration on the discharge capacity indicates significant hindrance in the transport of SO_2_ to the cathode, even with a great surplus of unreacted SO_2_.

#### 2.1.2 Diagnosis of Low SO_2_ Utilization

**Figure 1:**
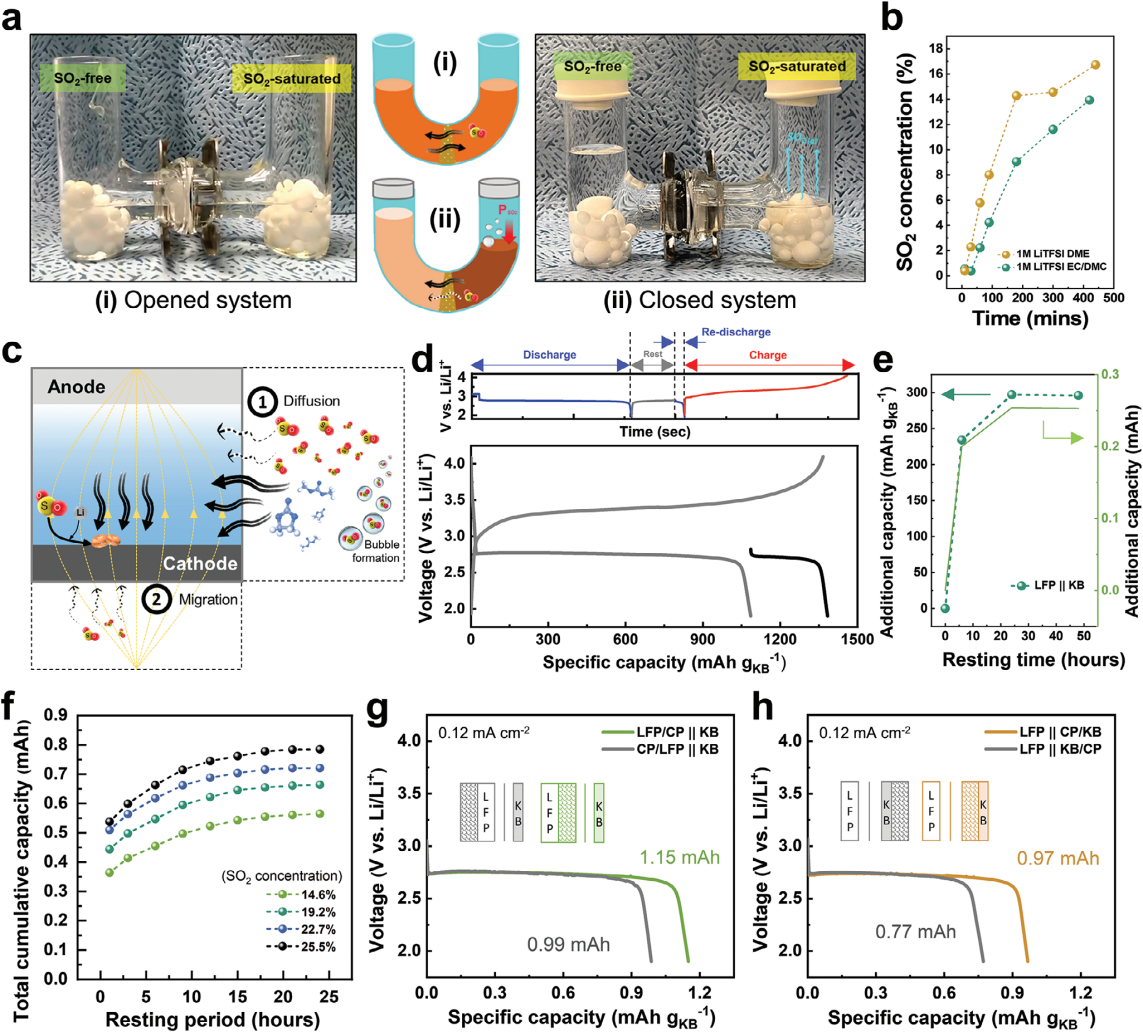
Qualitative and quantitative analysis of the diffusion behavior of SO_2_ within a cell stack. a) Schematic and digital images for assessment of SO_2_ diffusion in H‐cells with i) open and ii) closed ends. b) Concentration evolution of SO_2_ in the SO_2_‐free container (i.e., left container in H‐cells) over time in two different electrolytes. c) Schematic diagram depicting the influence of diffusion and migration on the movement of SO_2_. d) Charge‐discharge curves of an LFP||KB cell with a resting period of 24 h at room temperature following complete discharge. e) Additional capacity as a function of the length of the resting period after complete discharge. f) The total cumulative discharge capacity of Li||KB cells with different SO_2_ concentrations in the electrolyte as a function of the length of the resting period after complete discharge. Discharge curves for cells with CP inserted at different locations in the cell either on g) the LFP counter electrode side or on h) the KB cathode side. The insets in (g,h) are schematics of the arrangement of electrodes in each cell.

To understand the transport kinetics of SO_2_, we employed an H‐shaped cell commonly used for osmosis studies (**Figure** **1**a). The left compartment contains 1 m LiTFSI EC/DMC (1:1 v/v) electrolyte, while the right is filled with the electrolyte saturated with SO_2_, reaching an SO_2_ concentration of 25.5 wt%. These compartments are divided by a glass microfiber filter (GF/F), facilitating the unrestricted diffusion of solutes and solvents. When both tubes are open to the ambient, the liquids on both sides remain at their original levels (Figure 1a(i)). When both tubes are sealed, however, the level of the SO_2_‐free side is significantly higher. Apparently, organic solvents are driven by osmotic effect to the side with higher lithium salt concentrations, rather than SO_2_ being driven to the left by concentration gradient. As a result, the concentration of SO_2_ exceeds the saturation limit on the right, leading to a buildup of pressure that provides the balance observed in Figure 1a(ii). This observation can be explained by viewing SO_2_ as a less competitive solvent for lithium salt than organic solvents.^[^ ^15^ ^]^ Further, diffusion of SO_2_ is apparently slow which is quantified by measuring the SO_2_ concentration in the left compartment as a function of time (Figure 1b). Regardless of electrolyte type (e.g., carbonate‐ and ether‐ based electrolytes), equilibrium of the SO_2_ concentration is not achieved even after many hours. This observation indicates that in a coin cell, once the SO_2_ is consumed in the electrolyte volume between the anode and the cathode, organic solvents rather than SO_2_ will be driven by osmotic forces to enter the cell stack volume, leaving SO_2_ behind. This transport dynamic thus limits the utilization of SO_2_.

#### 2.1.3 Approaches to Improve SO_2_ Utilization in Coin Cells

As schematically shown in Figure 1c, the transport of SO_2_ in a sealed system involves: 1) diffusion driven by concentration gradient; and 2) migration induced by electric field force for SO_2_ molecules bonded to charged ions. We next quantify the relative contributions of these two transport mechanisms. Figure 1d,e investigates whether additional influx of SO_2_ can occur when the cell is given a rest period after full discharge. To exclude the influence of undesired side reactions at the lithium surface leading to SO_2_ consumption, we fabricate the cell using lithium iron phosphate (LiFePO_4_ or LFP) as the counter electrode. LFP is designed with an excess lithium capacity, supporting efficient SO_2_ utilization. The cell is fully discharged until reaching 2.0 V vs. Li/Li^+^, then re‐discharged after resting for 24 h under open circuit, delivering an additional capacity of 298.2 mAh g_KB_^−1^ (Figure 1d). Note that even at a total capacity of 1383.6 mAh g_KB_^−1^, the KB cathode is far from being fully utilized. More extended resting does lead to higher capacities (Figure 1e). However, the overall utilization of SO_2_ is still only 14.6% after resting for 48 h. Furthermore, electrolytes with different starting concentrations of SO_2_ all show similar behavior, i.e., resting leads to additional capacities but the utilization remains low even after resting for 24 h (Figure 1f). To further probe the effect of electrolyte volume between the anode and the cathode, we attach a carbon paper (CP) either behind or in front of the LFP or KB electrode. Figure 1g shows the configuration with CP adjacent to LFP, whereas in Figure 1h, CP is positioned adjacent to KB (see the inserted schematics in Figure 1g,h). When comparing discharge capacities based on CP positions, it is evident that greater discharge capacities are achieved when CP is situated within the current path, irrespective of its adjacency to either electrode. Thus, CP does not serve as a reaction site but simply allows for more SO_2_ to be held between the cathode and anode by providing additional pore volume within the cell stack, resulting in higher discharge capacities.

### 2.2 Lithium Protective Layer for Long‐Term Cycling and SO_2_ Placement in the Current Path

#### 2.2.1 Introducing a Lithium Protective Layer

**Figure 2:**
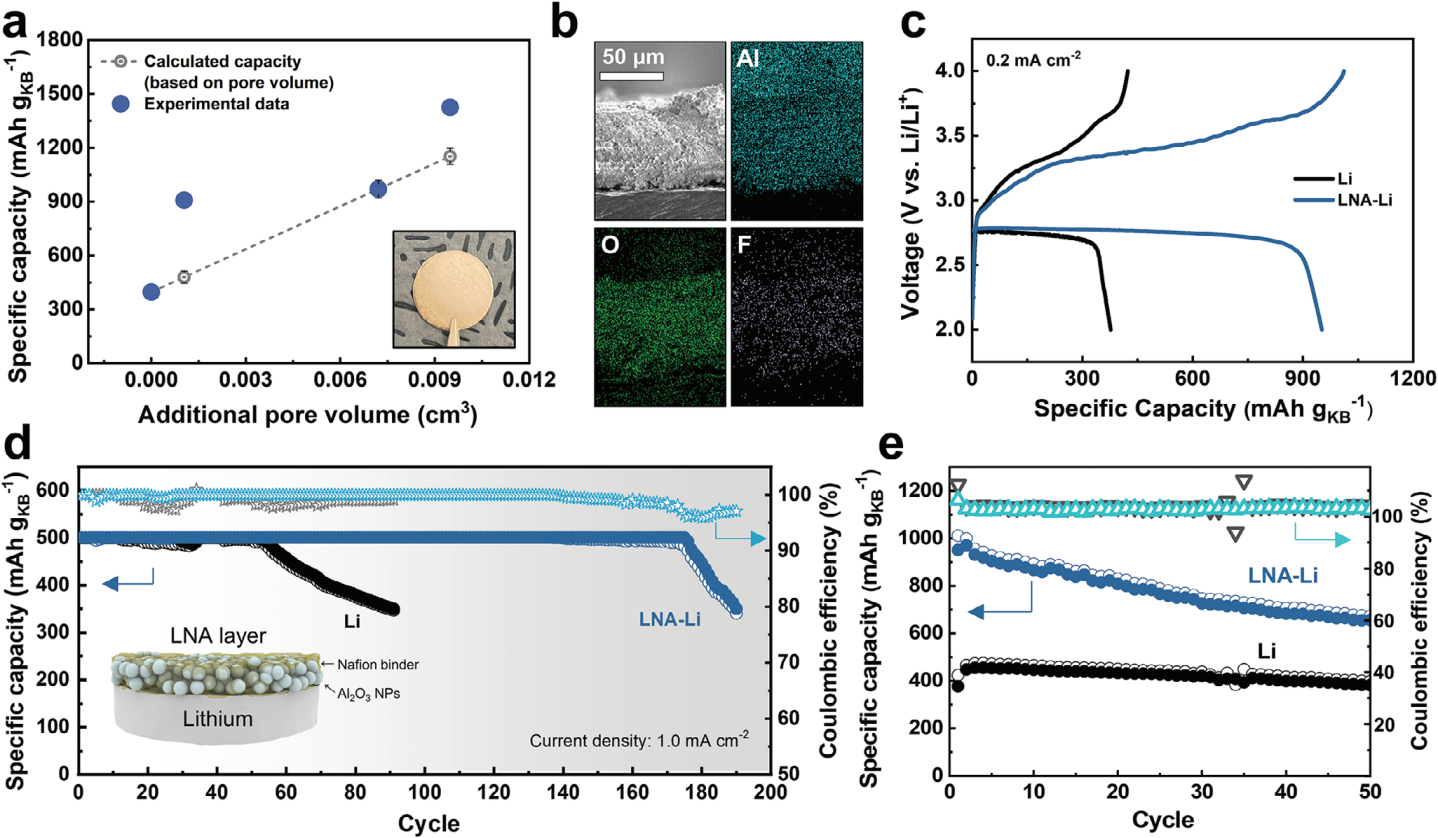
Improvement of capacity and cycling stability through a porous lithium protective layer. a) Specific capacity as a function of pore volume created by the porous protective layer. The inset in a) is a digital image of LNA‐Li. b) Cross‐sectional SEM and elemental mapping images of LNA‐Li. c) Charge–discharge curves of Li||KB cells with cell stacks, either with or without the lithium protection layer. d) Comparison of cycling characteristics using a limited capacity of 500 mAh g_KB_^−1^ until the voltage range of 2.0–4.0 V is reached. e) Cycling stability using a voltage range of 2.0–4.0 V at a current density of 0.2 mA cm^−2^.

Our next objective is to reduce the parasitic reaction between SO_2_ and lithium since both of them are limited in a sealed cell, in contrast to previously reported open systems where both reactants are in great excess. In this regard, we develop a protective layer consisting of lithiated Nafion and alumina nanopowders (Al_2_O_3_ NPs).^[^ ^23^, ^24^ ^]^ The integration of Al_2_O_3_ and lithiated Nafion provides exceptional mechanical strength and chemical stability, along with elevated ionic conductivity, making it a highly suitable coating material. Other oxide nanoparticles, such as SiO_2_ and ZrO_2_ might also be suitable. Compared to other polymers, lithiated Nafion shows improved ionic conductivity, enabling a low nucleation overpotential associated with lithium deposition (Figure S4, Supporting Information). By modifying the weight ratio of two solid components in the coating slurry, we fabricated coatings with thickness of 14.2, 70.2, and 94.6 µm (Figure S5, Supporting Information). With an increase in the coating thickness, the pore volume within a cell stack proportionately increases. As expected, this coating layer thus absorbs electrolyte and acts as a source of SO_2_ in the current path, resulting in increased discharge capacity (**Figure** **2**a). Elemental mapping images of Al, O and F indicate a homogeneous distribution of Al_2_O_3_ NPs and lithiated Nafion in the depth direction of the lithium protective layer (Figure 2b). The crack‐free coating, deposited with a thickness of 70 µm (denoted as LNA‐Li) is also evaluated in Li||Li symmetric cells. At a current density of 0.5 mA cm^−2^, the cells exhibited an overpotential 91.0 mV higher than bare lithium, due to the thickness of the coating layer (Figure S6, Supporting Information). When the same coating is implemented in a Li–SO_2_ coin cell, the observed capacity is ≈2.5 times that of the control cell without the coating layer (Figure 2c).

To evaluate the effect of the coating layer on the cycling stability of Li–SO_2_ cells, we employ two different cycling protocols. Figure 2d shows the capacity retention when the discharge capacity is fixed at 500 mAh g_KB_^−1^ until the cell exceeds its prescribed voltage limit of 2.0–4.0 V vs. Li/Li^+^. The cell incorporating LNA‐Li lasts 3.3 times longer than the cell with bare lithium. We also cycled the cells directly between 2.0–4.0 V at a current density of 0.2 mA cm^−2^ (Figure 2e). The cell with LNA‐Li consistently shows significantly higher capacity than the control. To demonstrate that the effectiveness of this layer is not solely due to its ability to contain more SO_2_ within the current path, we evaluated the cycle stability of cells using 420 µm thick glass fiber (GF/F) membranes (Figure S7, Supporting Information). Although GF/F separators can absorb more electrolyte due to their larger pore volume, they showed significantly poorer cycle performance compared to cells with LNA‐Li. This highlights the effectiveness of the LNA coating in mitigating the irreversible depletion of SO_2_ due to its reaction with lithium. The exposure of fresh lithium surfaces during cycling promotes the formation of a solid electrolyte interphase (SEI) in the SO_2_‐containing electrolyte, which consists of Li‐S‐O reduction products and leads to the irreversible consumption of SO_2_.^[^ ^23^ ^]^ Thus, controlling lithium growth to minimize surface area is key to reducing SO_2_ loss. In the case of LNA‐Li, lithium growth occurs beneath the dense layer of the LNA coating, effectively inhibiting dendritic growth (Figure S8, Supporting Information) and minimizing the exposure of new surfaces (Figure S9, Supporting Information).

### 2.3 A study on Enhancing SO_2_ Reversibility Using Electrolyte Additives

#### 2.3.1 Improving the Reversibility of SO_2_ Redox with TPA

The reversibility of the SO_2_ discharge products plays a crucial role in conserving the total amount of active SO_2_. The discharge product, Li_2_S_2_O_4_, readily undergoes disproportionation to transform into the highly insulating solid Li_2_SO_4_ and elemental sulfur (S), hindering the reversible utilization of SO_2_ (Equation (1)).

(1) $$Li_{2}S_{2}O_{4}\left(s\right)\to Li_{2}SO_{4}\left(s\right)+S\left(s\right)$$

**Figure 3:**
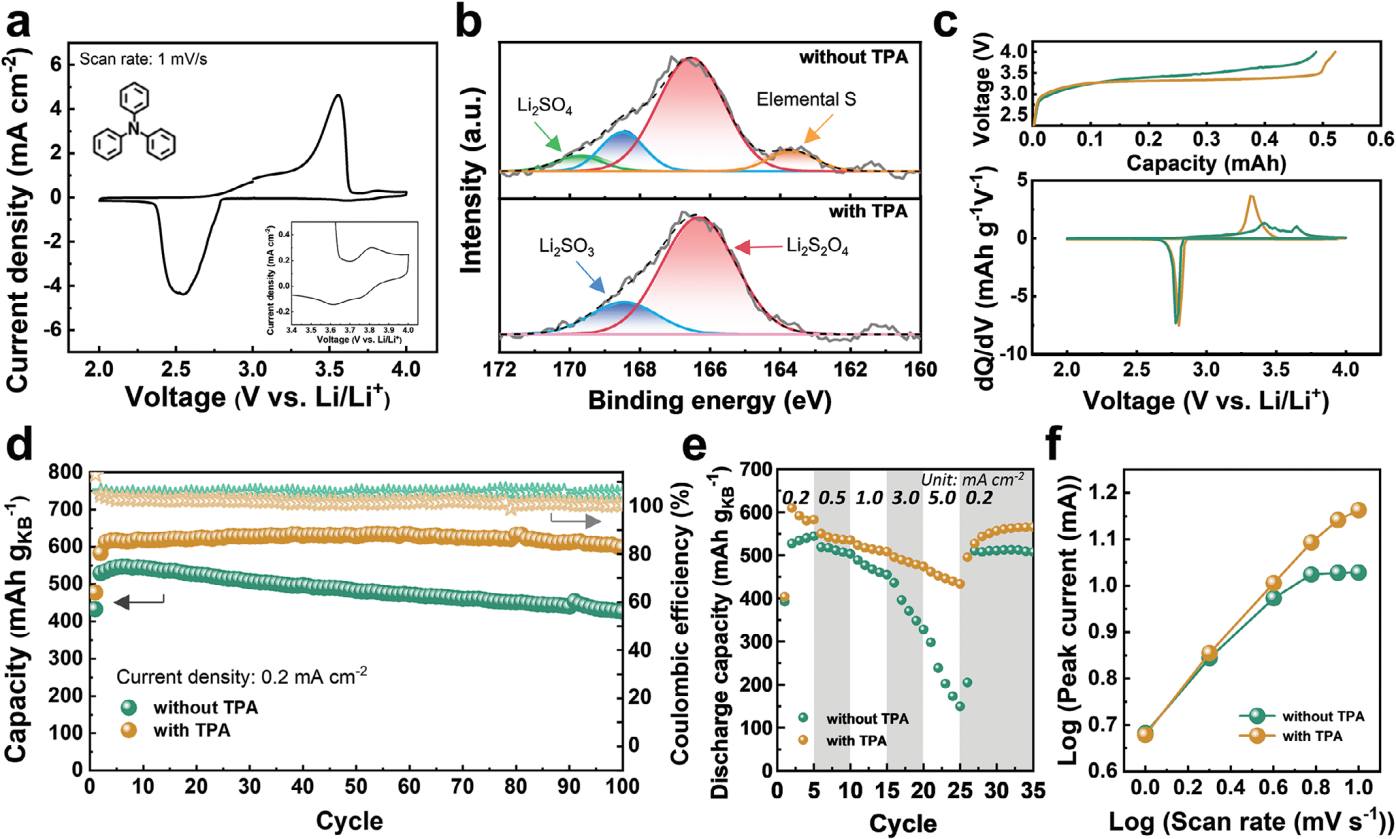
Accelerated electrochemical dynamics and alleviation of lithium side reactions through the electrolyte additive of TPA. a) CV curves of SO_2_‐saturated carbonate electrolyte with TPA additive. The inset in a) is the structure for a TPA molecule and a magnified portion between 3.4–4.0 V. Comparison of b) XPS patterns for Li||KB cells with and without TPA additive after discharge and c) differential capacity (dQ/dV vs. V) curves (lower) corresponding to charging curves (upper). d) Cycling stability of Li||KB cells between 2.0–4.0 V. e) Rate capability comparison; and f) Anodic peak current as a function of scan rates.

Triphenylamine (TPA) has the potential to prevent S oxidation by supplying electrons to the unstable Li_2_S_2_O_4_ due to its electron‐rich nature (refer to inset of **Figure** **3**a for chemical structure).^[^ ^13^ ^]^ First, we evaluate the stability of TPA in SO_2_‐containing electrolyte. (Figure 3a). The reversible redox behavior of TPA/TPA^+^ is observed at 3.5–4.0 V vs. Li/Li^+^. That is the region where the oxidation peak of SO_2_ is 3.65 V. A more detailed view is shown in Figure S10 (Supporting Information). We note that TPA has an insignificant but stable contribution to the capacity as confirmed by evaluating cells without SO_2_ (Figure S11, Supporting Information). Additionally, X‐ray diffraction (XRD) analysis (Figure S12, Supporting Information) confirms that the introduction of TPA does not change the discharge product of the Li–SO_2_ chemistry.^[^ ^1^, ^29^ ^]^ To examine the chemical reactivity of TPA on the lithium metal anode, we immersed bare lithium in EC/DMC (1:1 v/v) solvent with and without TPA for 24 h at room temperature. Analysis of the resulting surface morphology and SEI composition (Figures S13 and S14, Supporting Information) revealed that the SEI, primarily composed of Li_2_O and Li_2_CO_3_, was unaffected by the presence of TPA, indicating negligible reactivity between TPA and lithium.^[^ ^30^, ^31^ ^]^ Moreover, considering the reversibility of TPA and the role of the LNA coating layer in mitigating reactions between soluble SO_2_ and lithium, the effect of the low concentration TPA^+^ on the lithium surface is expected to be negligible.

To unravel the role of the TPA catalyst, X‐ray photoelectron spectroscopy (XPS) analysis of the cathode is conducted to examine the discharge products (Figure 3b). Predominant peaks corresponding to Li_2_S_2_O_4_ and Li_2_SO_3_ at 166.5 and 168.6 eV, respectively, are observed, confirming the prevalence of reversible discharge products in both cases, with or without TPA.^[^ ^8^ ^]^ However, in the absence of TPA, the cathode displays additional peaks at 169.0 and 164.0 eV, signaling the presence of Li_2_SO_4_ and elemental S. These compounds are identified as disproportionation products derived from Li_2_S_2_O_4_. As shown in Figure 3c, the catalyst reduces the charging overpotential. This is clearly indicated by the SO_2_ oxidation peak appearing at a lower voltage of 3.33 V vs. Li/Li^+^ in the corresponding differential capacity plots (dQ/dV vs V) (Figure 3c). Figure 3d shows the effect of TPA on the cycle life in Li||KB cells. The cell containing TPA maintains 97.6% of the initial capacity after 100 cycles, significantly higher than the cell without TPA. Similar beneficial effects are also observed when the cells are cycled with a limiting capacity of 500 mAh g_KB_^−1^ (Figure S15, Supporting Information). Ultimately, the combination of TPA with LNA‐Li enhances the cyclic stability, while also allowing the cathode to achieve a capacity of 1023.3 mAh g_KB_^−1^ (Figure S16, Supporting Information). Moreover, TPA also boost the rate performance of the reaction (Figure 3e; Figure S17, Supporting Information). When the current density is increased from 0.2 to 5.0 mA cm^−2^, a significant capacity reduction to 27.5% occurs without the catalyst, whereas with TPA, it is sustained at 74.5%.

Finally, we also assessed the reaction kinetics using CV measurements (Figure S18, Supporting Information). We plot log(peak current) against log(scan rate) and compare the values of the slopes (Figure 3f).^[^ ^32^ ^]^ Within the range of 2.0–4.0 mV s^−1^, the curve exhibits a linear relationship for both electrolytes. However, beyond a scan rate of 6.0 mV s^−1^, the absence of a catalyst leads to a lack of further increase of currents, indicating difficulties in charge transfer. In contrast, cells with TPA‐containing electrolytes demonstrated much‐improved discharge kinetics. The reaction kinetics further influence the morphology of discharge products (Figure S19, Supporting Information).^[^ ^33^, ^34^ ^]^

### 2.4 Proposed Cell Format for Increasing SO_2_ Utilization

#### 2.4.1 Bobbin‐Cell Demonstration

**Figure 4:**
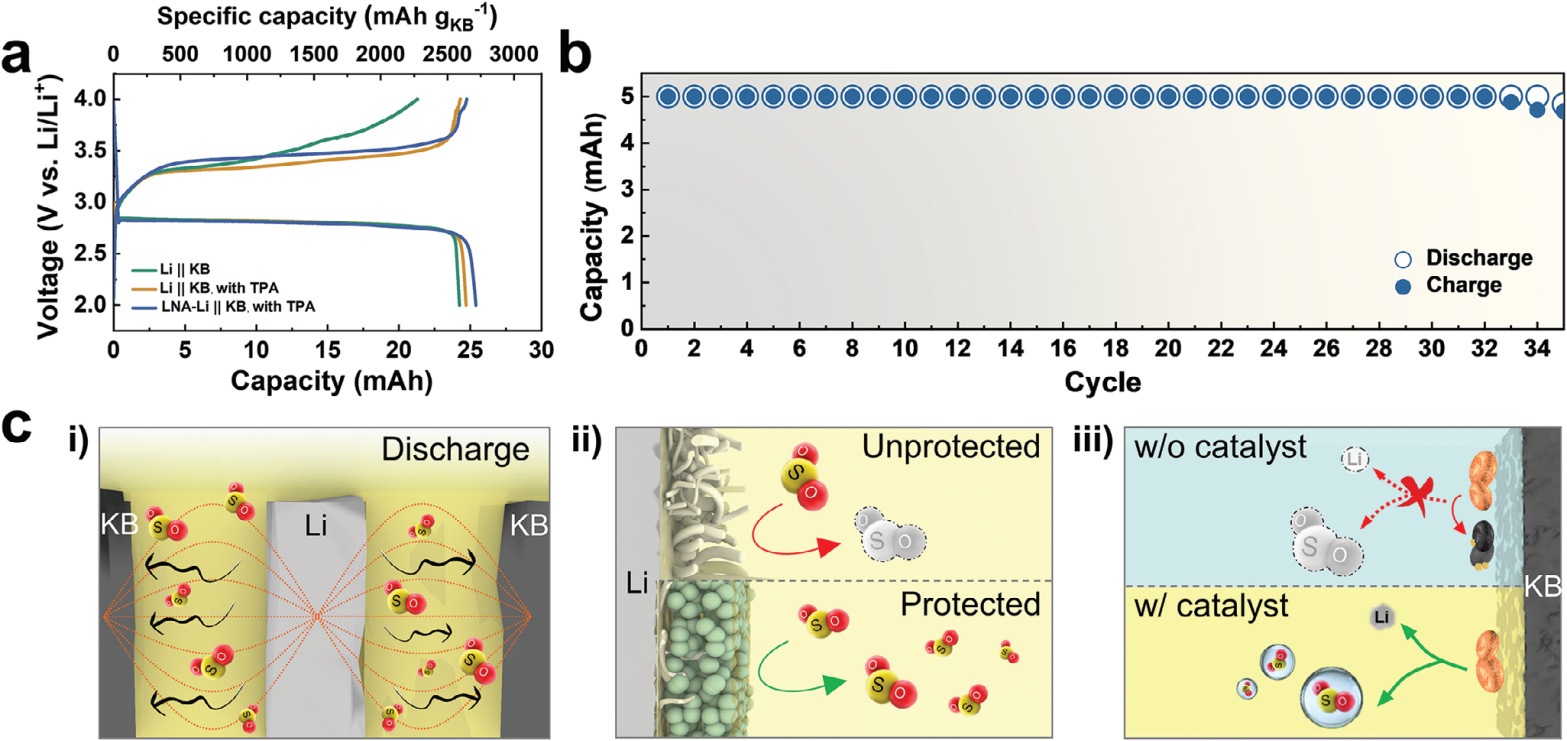
A Proof of concept demonstration using a bobbin‐type cell. a) Charge‐discharge curves of bobbin‐cells containing different counter electrodes (bare Li or LNA‐Li) and electrolytes (with or without TPA) at current density of 0.2 mA cm^−2^. b) Cycling characteristics with a fixed capacity of 5.0 mAh at a current density of 1.0 mA cm^−2^ up to the voltage range of 2.0–4.0 V. c) Summary of the design principles for a sealed type Li–SO_2_ battery; i) Placement of all electrolytes within a cell stack. Introduction of ii) a lithium protective layer and iii) an electrolyte additive.

Our understanding of the transport kinetics of SO_2_ favors a cell design where all electrolytes are housed between its two electrodes, similar to a bobbin‐cell.^[^ ^35^ ^]^ A proof of concept demonstration is shown in Figure S20 (Supporting Information). The KB cathode and lithium anode are arranged in a “donut‐like” configuration within a culture tube (Figure S21, Supporting Information). This configuration, where all SO_2_ is confined between the two electrodes, maximizes SO_2_ utilization and mitigates the effect of intrinsically slow diffusion kinetics of SO_2_ from outside the current path. We assembled the cell using 0.354 g of SO_2_‐saturated electrolyte (expected capacity of 37.8 mAh at full utilization), KB electrodes with a loading of 3.3 mg cm^−2^ and a 200 µm thick lithium foil as a counter electrode positioned at the center. The three cells (with or without TPA and/or LNA coating layer) show similar discharge capacities of 24.2 to 25.4 mAh. This indicates 67.2% utilization of SO_2_, which is 12.7 times higher than in coin cells (**Figure** **4**a; Table S2, Supporting Information). The comparable SO_2_ utilization achieved irrespective of the presence of the LNA coating layer indicates that the bobbin‐cell configuration offers a favorable arrangement for SO_2_. The coulombic efficiency values of 87.9%, 98.4%, and 97.5% further emphasize the critical role of the TPA catalyst and protective layer in enhancing the reversibility of SO_2_ redox reactions.

Figure 4b demonstrates the cycling stability of the cell, achieved by incorporating the proposed strategies, with a limiting capacity of 5.0 mAh at a current density of 1.0 mA cm^−2^. The cell exhibited stable performance for over 30 cycles. Additionally, we evaluated the performance of a Swagelok‐type cell with LNA‐Li and TPA in a closed system (Figure S22, Supporting Information). This configuration only achieved ≈25% SO_2_ utilization and a cumulative capacity 55.1% of that observed in the bobbin‐type cell, despite having the same areal capacity. This finding highlights the advantages of the bobbin‐cell design, particularly regarding the spatial positioning of SO_2_ within the cell stack.

Figure 4c schematically summarizes the design principles for a sealed Li‐SO_2_ battery. By employing a simple bobbin‐type cell, we i) maximize SO_2_ utilization through the placement of all redox‐active electrolytes within the cell stack. Additionally, we minimized the irreversible consumption of SO_2_ due to side reactions by applying an ii) LNA coating and iii) TPA additives. This approach enabled us to transition from the conventional coin cell to the bobbin‐cell, achieving a calculated energy density of 151.2 Wh kg^−1^. The calculation was derived from the experimentally obtained capacities and the actual weights of the electrodes used (Figures S23 and S24 and Table S3, Supporting Information). With further optimization, mainly using a light‐weight current collector, we project a cell energy density of 183.2 Wh kg^−1^ based on the energy density model described in Note S2 (Supporting Information). This energy density projection assumes an N/P of 2 and redox‐inactive components (current collectors, separators, coating layer) constituting <3% of the total weight.^[^ ^36^, ^37^ ^]^ We also show in Note S3 (Supporting Information) a possible design of a cell in a commercially relevant cylindrical format.

## 3 Conclusion

In order to develop a sealed Li–SO_2_ battery operating without external SO_2_ supply, we have systematically studied the SO_2_ transport kinetics. The osmotic behavior of the electrolyte necessitates the placement of all electrolytes directly in the current path to realize high SO_2_ utilization. The use of an electron‐rich TPA catalyst enhances reaction kinetics and alleviates discharge product disproportionation, suppressing the generation of irreversible insulating products. The integration of a lithium protective layer, alongside this electrolyte additive, promotes SO_2_ reversibility. In a bobbin‐cell configuration, close to 70% utilization of SO_2_ is achieved with a coulombic efficiency of 98%. This cell configuration results in a calculated energy density of 151.2 Wh kg^−1^. Our work illustrates the viability of sealed rechargeable Li–SO_2_ batteries for practical applications by leveraging the existing SO_2_ handling infrastructure.

## 4 Experimental Section

### Preparation of SO_2_‐Containing Electrolyte

Ketjen black carbon (KB, EC 600JD, MSE Supplies) was dispersed with a polytetrafluoroethylene (60 wt% dispersion, Teflon 30B, Polysciences) binder in a mass ratio of 9:1 into a solution of isopropanol (> 99.5%, ACS regent, Sigma‐Aldrich), N‐methyl‐2‐pyrrolidone (NMP, >99.5%, anhydrous, Sigma‐Aldrich) and water with a volume ratio of 23:5:20. The KB cathode was fabricated by casting the carbon paste on the carbon‐coated Al foil current collector and dried overnight at 110 ⁰C to evaporate the solvent and residual water. The average mass loading of the KB electrodes with a 12 mm diameter was 0.8–0.9 mg cm^−2^. Lithium iron phosphate (LFP, TCI Chemicals) was mixed with polyvinylidene fluoride (HSV1800, Kynar), Graphite (KS6, CPreme), and Super P in a mass ratio of 75:10:10:5 into an NMP solution. The prepared LFP paste was casted on the Cu foil current collector and dried overnight under vacuum. The average loading mass of the LFP electrodes with a 14 mm diameter was ≈29.25 mg cm^−2^. Electrolytes of 1 m lithium bis(trifluoromethane)sulfonimide (LiTFSI, TCI Chemicals) dissolved in ethylene carbonate/dimethyl carbonate (EC/DMC, Gotion) at a 1:1 volume ratio and 1 m LiTFSI in 1,2‐dimethoxyethane (DME, Gotion) were used as the baseline electrolyte. SO_2_ gas was injected into the closed vial with a rubber stopper using needles. The gas was injected at a pressure of 15 psi for durations of 5, 10, 20, and 40 min until saturation was reached. Triphenylamine (TPA, 98%, Sigma‐Aldrich) was first dissolved into the baseline electrolyte to reach a concentration of 20 mm. SO_2_ was then bubbled into this solution. After saturation, the outlet hole was opened for 1 min to eliminate residual SO_2_ gas in the head space of the vial.

### Preparation of Li–SO_2_ Cells (Coin‐ and Bobbin‐Type)

CR2032 Li–SO_2_ coin cell was assembled by sequentially stacking lithium metal with a diameter of 13 mm, one sheet of separator (Celgard 3401, Celgard) with a diameter of 19 mm, and the prepared carbon electrode with a diameter of 12 mm in an Ar‐filled glove box (O_2_ and H_2_O level <1 ppm). The amount of electrolyte was 0.076 g. The bobbin‐type cell was built by placing a culture tube (10×75 mm, 4 ml, Pyrex 9820 Borosilicate glass round bottom, Corning) with an inner diameter of 8 mm and a glass stirring rod with a diameter of 6 mm in the middle. The Cu mesh foil was cut into 2 cm by 2 cm pieces and welded with Ni tabs. The KB electrode, which was cast onto stainless‐steel gauze (200 mesh woven from 0.05 mm dia. wire, Type 316, Thermo Scientific) was cut into 1.3 cm by 2.4 cm pieces and welded with Al tabs. The cathode surface was fully covered using a separator and battery strapping tape, then positioned against the inner wall of a culture tube. The Cu mesh was wound around a glass rod. Lithium, with a thickness of 200 µm and dimensions of 2.0 cm by 1.5 cm, was securely attached on the Cu mesh foil in an Ar‐filled glove box. A total of 0.354 g of SO_2_‐containing electrolyte was introduced into the tube housing the cathode. Subsequently, the glass rod with lithium was aligned within the tube, and the cell was sealed with rubber O‐rings to prevent SO_2_ gas leakage. The assembled bobbin‐cells were electrochemically evaluated within an airtight enclosure.

### Assembly of H‐Cell and SO_2_ Titration

A glass microfiber filter (grade GF/F, Whatman) was fastened with O‐rings and a clamp in the center of an H‐shaped electrochemical cell. SO_2_‐free and SO_2_‐saturated electrolytes were poured simultaneously into the left and right compartments, respectively. The H‐cell was promptly sealed using rubber stoppers. At regular intervals, electrolytes were extracted from the left compartment (i.e., SO_2_‐free container) using a syringe for SO_2_ titration. The SO_2_ titration was performed to determine the SO_2_ concentration of electrolytes over time. A diluted 5% H_2_O_2_ (30%, Fisher chemicals) and a 5 m NaOH (28‐30%, ACS reagent, Sigma‐Aldrich) solutions were prepared. Two drops of a mixed indicator solution containing Methylene blue and methyl red dissolved in ethanol (Methyl red–Methylene blue solution, TCI Chemicals) were added to the H_2_O_2_ solution. Next, 1 ml of the extracted electrolyte was mixed with 4 ml of the H_2_O_2_ solution. Then, the NaOH solution was gradually added in increments of 5 µl until the solution changes color from purple to green. The SO_2_ concentration was calculated based on the volume of 5 m NaOH solution added.

### Preparation of LNA‐Li

A slurry was prepared by mixing lithiated Nafion, alumina nanopowders (Al_2_O_3_ NPs, particle size <50 nm, Sigma‐Aldrich), and dimethyl sulfoxide (DMSO, ≥99.9%, anhydrous, Sigma‐Aldrich). To lithiated Nafion, 25.2 mg of LiOH∙H_2_O (>98%, ACS reagent, Sigma‐Aldrich) was added to 10 ml of commercial Nafion solution (Nafion 117 solution, Sigma‐Aldrich). Subsequently, the resulting mixture was stirred at 60 °C for 2 h. The lithiated Nafion dispersion was dried in a vacuum oven (MTI oven) at 80 °C for 12 h to obtain the lithiated Nafion polymer as a solid residue. The prepared lithiated Nafion powder (30 mg) was dissolved in 1 mL of DMSO along with 150 mg of Al_2_O_3_ NPs, and the mixture was stirred overnight in an Ar‐filled glove box. The prepared slurry was drop casted on the lithium metal and then drying under inert atmosphere at room temperature for one day.

### Characterization of Li–SO_2_ Cells

All the electrochemical tests of the Li–SO_2_ cells were performed using a potentiostat (LBT‐5V5A battery tester, Arbin Instruments) between 2.0 and 4.0 V at room temperature. For the lithium symmetric cell tests, a coin‐type cell (CR2032) was assembled in the same way. Electrochemical impedance measurements (EIS) and Cyclic voltammetry (CV) were performed by using a potentio‐galvonostat (VSP‐300, Bio‐Logic Science Instruments). The frequency range for the EIS was from 7 MHz to 50 mHz. All experiments are performed at room temperature.

XRD spectra of the cathodes were collected on a powder diffractometer (XRD, Bruker D2 Phaser). The sample was sealed with Kepton under Ar atmosphere. The system used Cu Kα radiation (λ = 1.5418 Å, 40 kV, 40 mA), and the sample was scanned in a 2θ range from 10° to 80° at a scan rate of 1°/s. The morphology and elemental mapping were examined by Field Emission scanning electron microscope (SEM, JEOL JSM‐7400F) and Zeiss Sigma 500 scanning electron microscope. X‐ray photoelectron spectroscopy (XPS, AXIS Supra XPS, Kratos Analytical) was used for the surface chemical characterization of the cathodes in an Ar atmosphere without air exposure.

## Conflict of Interest

The authors declare no conflict of interest.

## Supporting information



Supporting Information

Supplemental Video 1

## Data Availability

The data that support the findings of this study are available from the corresponding author upon reasonable request.
