# Supplementary material for: Development of a Sealed Rechargeable Li–SO2 Battery
Source: Adv Sci (Weinh). 2024 Dec 17;12(6):2411598. doi: 10.1002/advs.202411598 (PMC11809408; doi:10.1002/advs.202411598)
Supplement: Supplementary file 1 — Supporting Information [file ADVS-12-2411598-s001.pdf]

ADVANCED SCIENCE
Open Access

Supporting Information

for *Adv. Sci.*, DOI 10.1002/advs.202411598

Development of a Sealed Rechargeable Li–SO_2 Battery

*Gayea Hyun, Myeong Hwan Lee, Haodong Liu, Shen Wang, Zeyu Hui, Victoria Petrova and Ping Liu**

Supporting Information

Development of a Sealed Rechargeable Li–SO_2 Battery

*Gayea Hyun, Myeong Hwan Lee, Haodong Liu, Shen Wang, Zeyu Hui, Victoria Petrova, Ping Liu**

Video S1; Electrolyte viscosity of 1 M LiTFSI EC/DMC a) without and b) saturated with SO_2
Figure S1 to S16
Table S1 to S3
Supporting Information Note 1 to 3

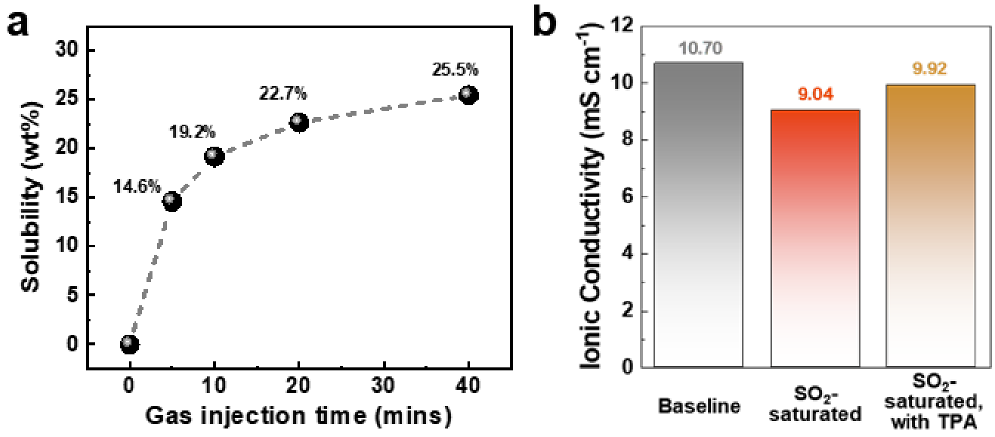

Figure S1. a) Solubility of SO_2 in 1 M LiTFSI EC/DMC (1:1 v/v) electrolyte as a function of SO_2 injection time. b) Comparison of ionic conductivities of baseline electrolyte (SO_2-free), SO_2-saturated electrolyte, and electrolyte containing SO_2 and soluble catalyst of triphenylamine (TPA).

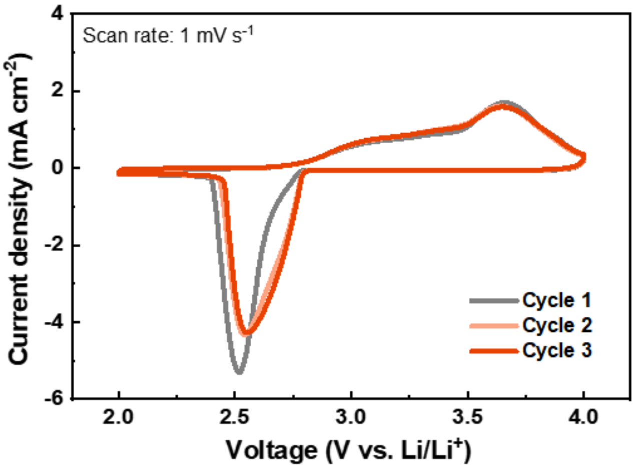

Figure S2. CV curves of the Li||KB coin cell with SO_2-saturated electrolyte. The scan rate is 1 mV s^{-1}.

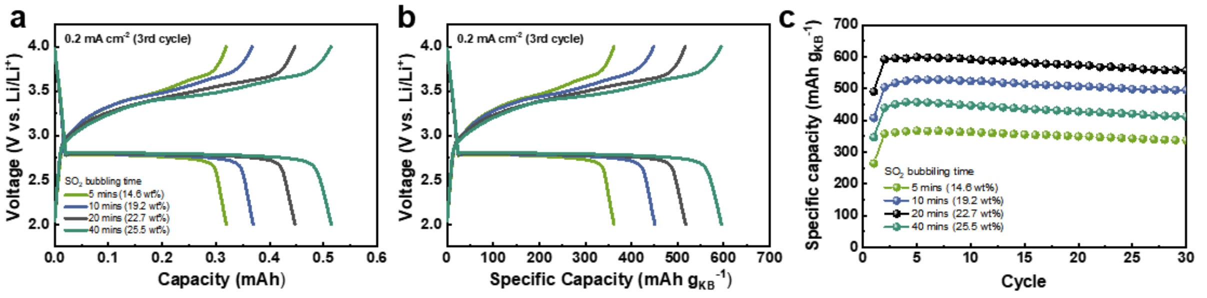

Figure S3. Charge-discharge curves for Li||KB cells containing electrolyte solutions with varying SO_2 concentrations: voltage as a function of a) cell capacity or b) specific capacity of the cathode (mAh g_{KB}^{-1}). c) Capacity retention during extended cycling.

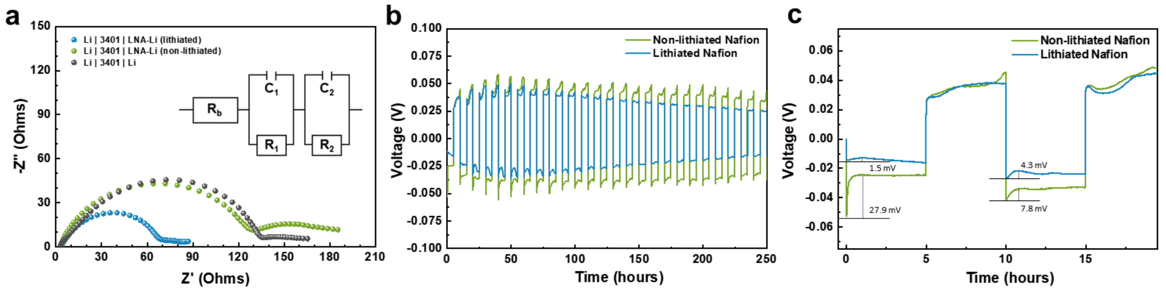

Figure S4. a) EIS curves prior to cycling for a Li||Li symmetric cells; Li||Li, Li||LNA-Li (with non-lithiated or lithiated Nation) (the inset depicts the equivalent circuit. R_b: bulk resistance, R_1: the resistance of the surface film, and R_2: charge transfer resistance). b) Voltage profiles during cycling for Li||Li symmetric cells containing either lithiated or non-lithiated Nafion in the LNA-Li layer, and c) an enlarged graph of the initial two cycles of the voltage curves. The cells were cycled at a current density of 0.2 mA cm^{-2} and a capacity of 1.0 mAh cm^{-2}.

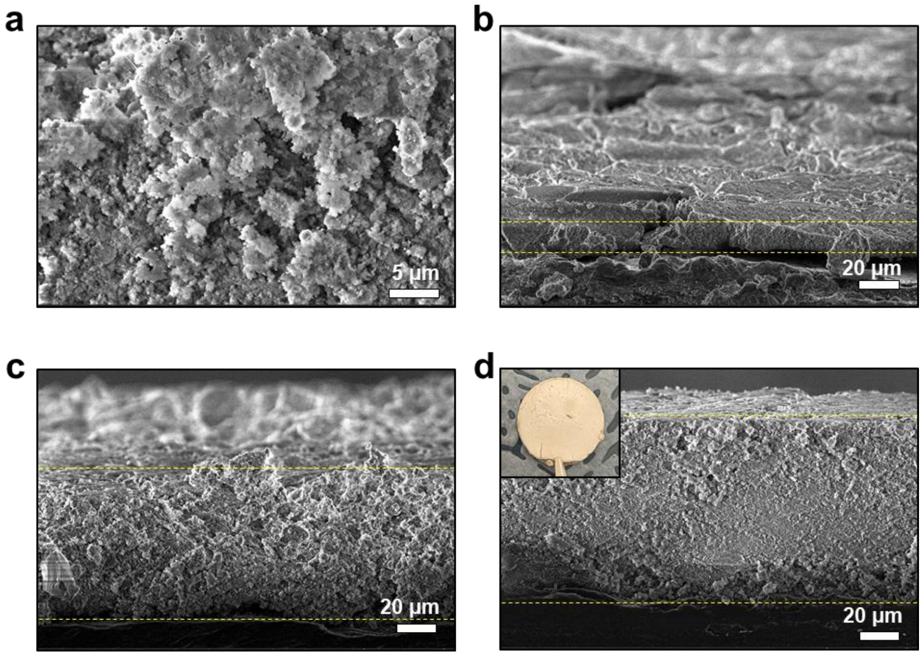

Figure S5. a) Top-view SEM image of a lithium protective layer. (b-d) Cross-sectional images of protective layers with different thicknesses prepared with increasing solid contents in the coating slurry. The inset in d) is a digital image of lithium coated with a protective layer.

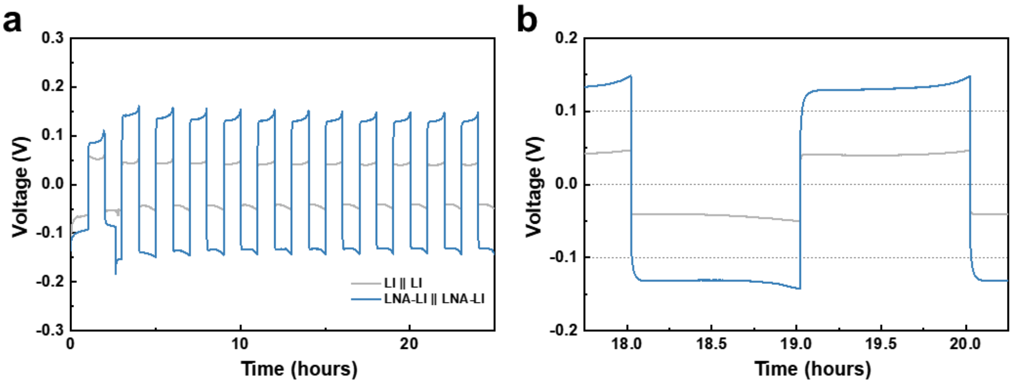

Figure S6. a) voltage curves of Li||Li symmetric cells cycling with a capacity of 0.5 mAh cm^{-2} at a current density of 0.5 mA cm^{-2}. b) a magnified portion of a).

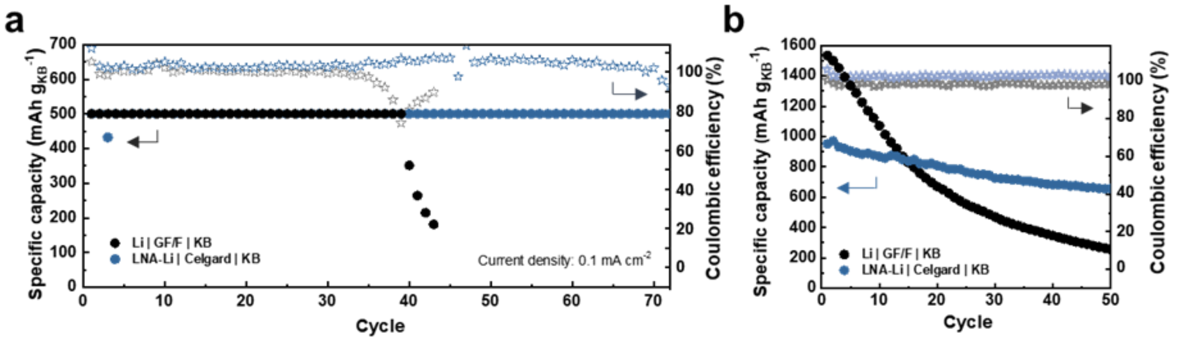

Figure S7. Comparison of cycling stability for cells with a GF/F separator or with an LNA coating on Li. a) Cycling is conducted with a limiting capacity of 500 mAh g^{-1} until the voltage range of 2.0–4.0V is reached. b) Cycling stability between 2.0–4.0V.

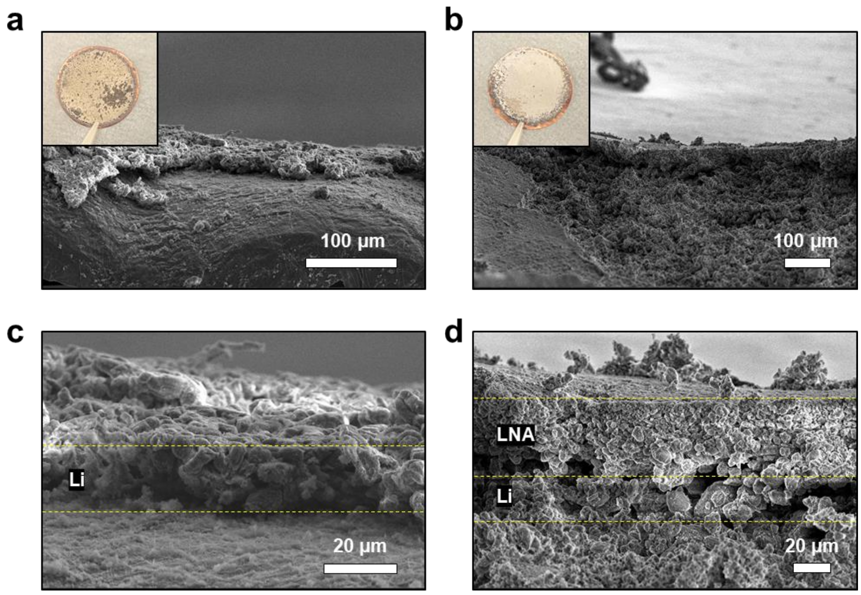

Figure S8. Observation of the deposited lithium in a Li||Li symmetric cell prepared with SO_2-containing electrolyte. Cross-sectional images of the electrode composed of a, c) bare Li and b, d) LNA-Li (inset images show digital photographs of the top surface of the lithium). Images c) and d) are magnified views of a) and b), respectively.

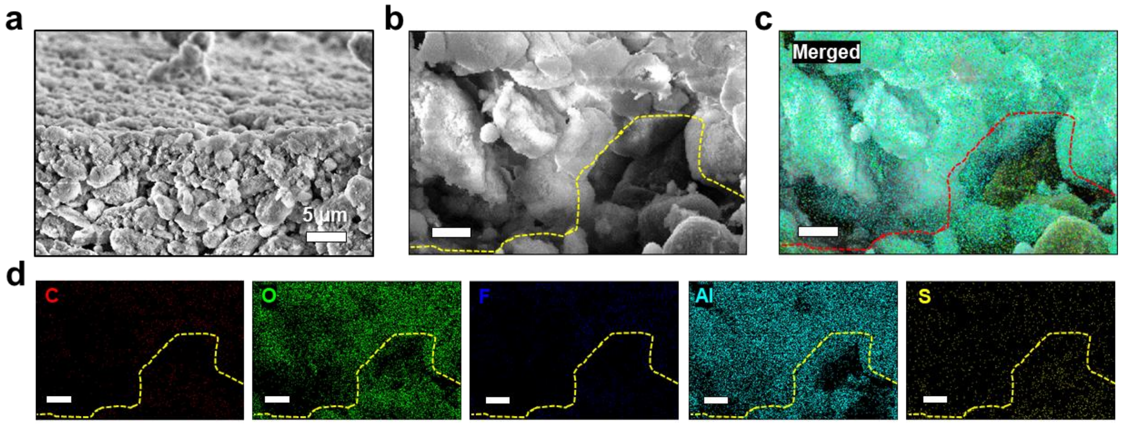

Figure S9. a) Magnified image of the top portion of the LNA coating in cycled LNA-Li from Figure S8. b) Enlarged view of the interface between lithium and the LNA coating layer, with corresponding EDX elemental analysis images. c) Merged image of the detected elements, and d) elemental mapping for C, O, F, Al, and S.

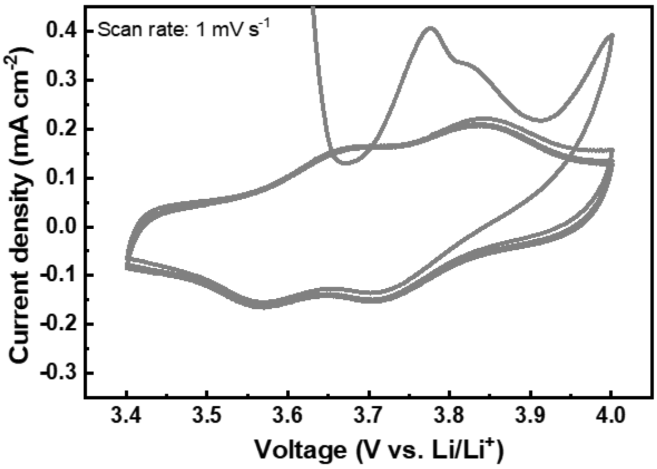

Figure S10. CV curves of SO_2-saturated electrolytes containing TPA between 3.4–4.0 V. The scan rate is 1 mV s^{-1}.

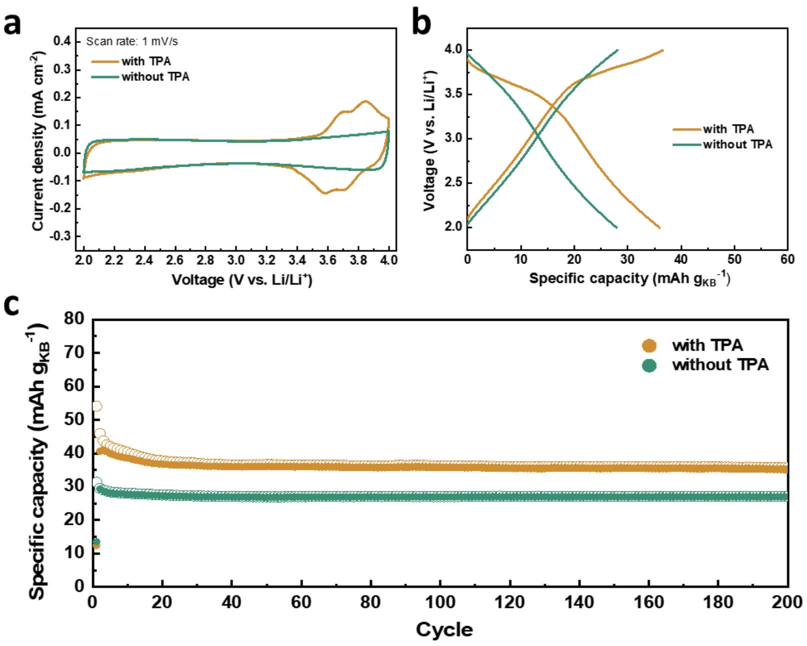

Figure S11. Electrochemical evaluation of a Li||KB cell in a 1 M LiTFSI EC/DMC (1:1 v/v) electrolyte with and without TPA. a) CV curves assessed at a scan rate of 1 mV s^{-1} within the voltage range of 2.0–4.0 V vs. Li/Li^+. b) Charge-discharge curves during the 20th cycle and c) cycling stability of Li||KB cells (solid circles: discharge; empty circles: charge).

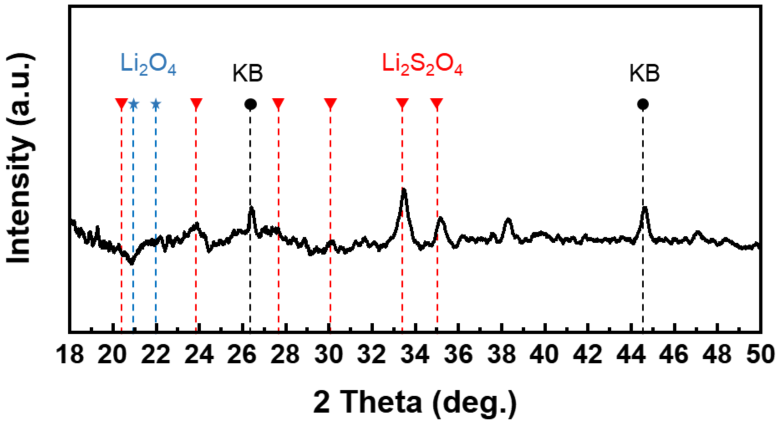

Figure S12. XRD profile of the KB cathode surface after cycling a cell containing SO_2-saturated electrolyte with TPA.

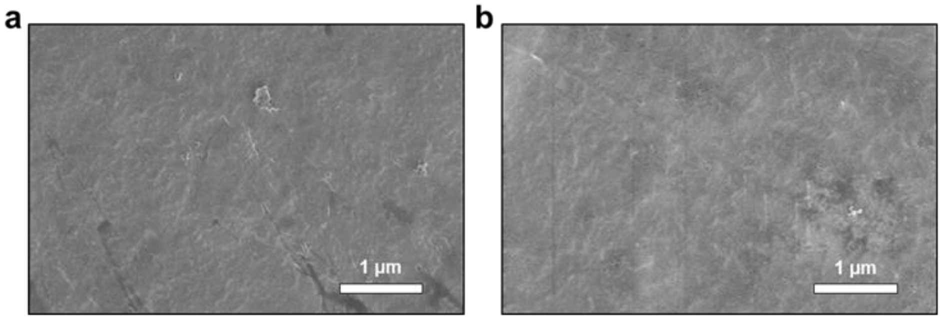

Figure S13. SEM images of bare lithium surfaces after immersion in EC/DMC (1:1 v/v) solvent at room temperature for 24 hours, a) without TPA and b) with 20 mM TPA.

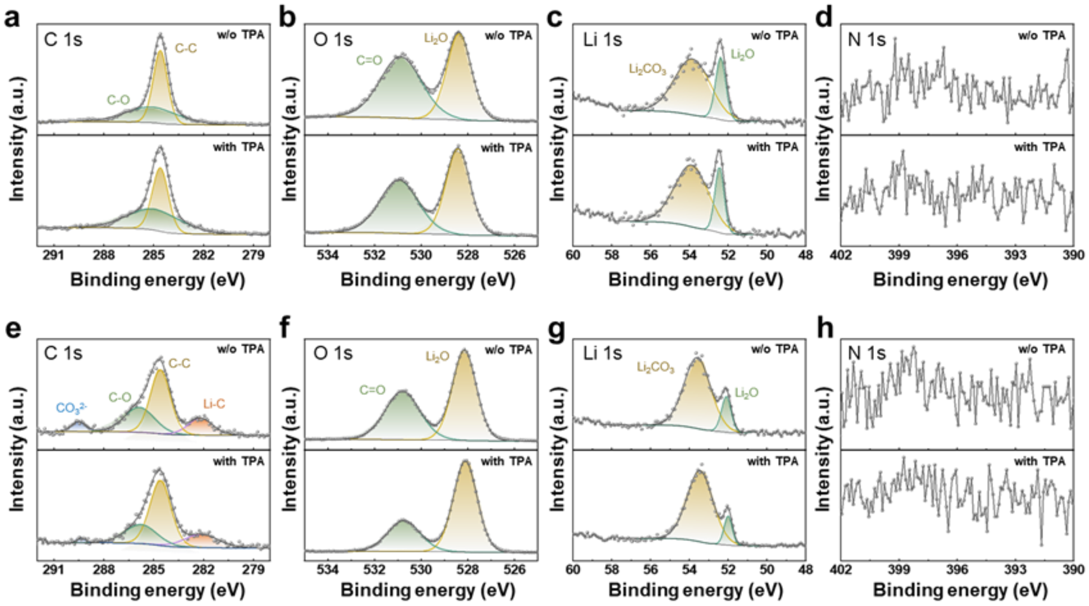

Figure S14. XPS spectra of bare lithium surfaces after immersion in EC/DMC (1:1 v/v) solvent at room temperature for 24 hours, either without or with TPA: a,e) C 1s, b,f) O 1s, c,g) Li 1s, and d,h) N 1s spectra. Panels a-d) show the surfaces before, and e-h) after, Ar cluster etching.

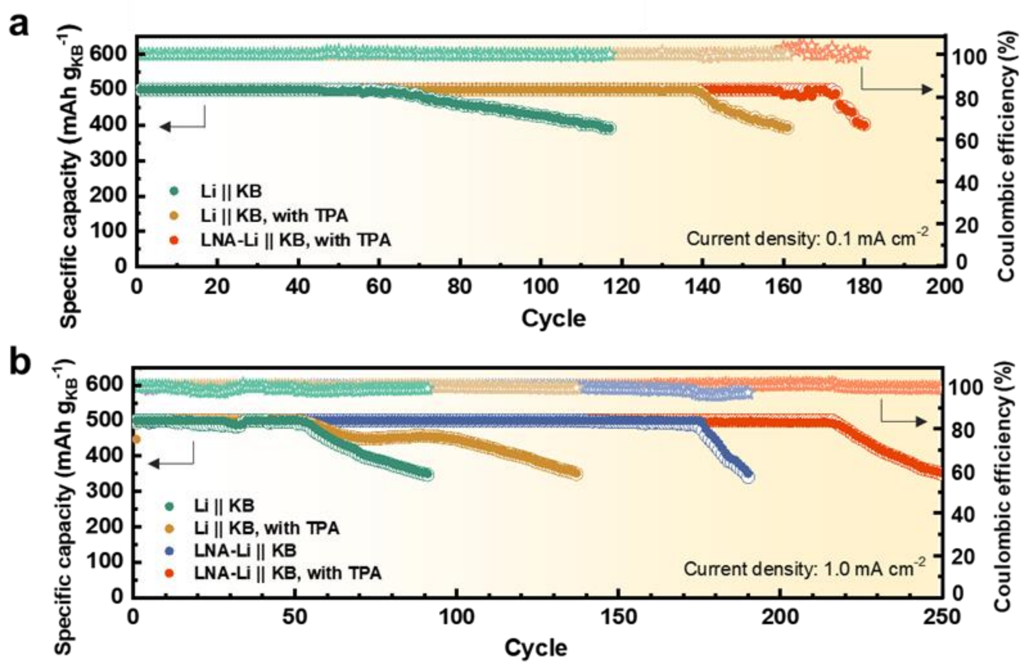

Figure S15. Comparison of cycling stability for Li||KB cells containing different counter electrodes (bare Li or LNA-Li) and SO_2-saturated electrolytes (with or without TPA) at the current density of a) 0.1 mA cm^{-2} and b) 1.0 mA cm^{-2}. The cycling is conducted with a limiting capacity of 500 mAh g^{-1} until the voltage range of 2.0–4.0V is reached (Solid circle: discharge, empty circle: charge).

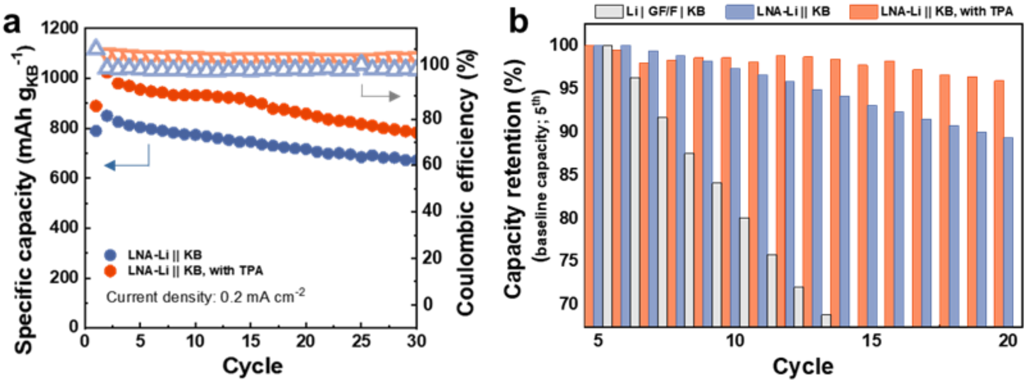

Figure S16. a) Cycling stability of a cell containing LNA-Li as a counter electrode, with SO_2-saturated electrolyte either with or without TPA. The current density is 0.2 mA cm^{-2}. b) Comparison of capacity retention for different cell configurations. The current density is 0.2 mA cm^{-2} and voltage range is 2.0–4.0V.

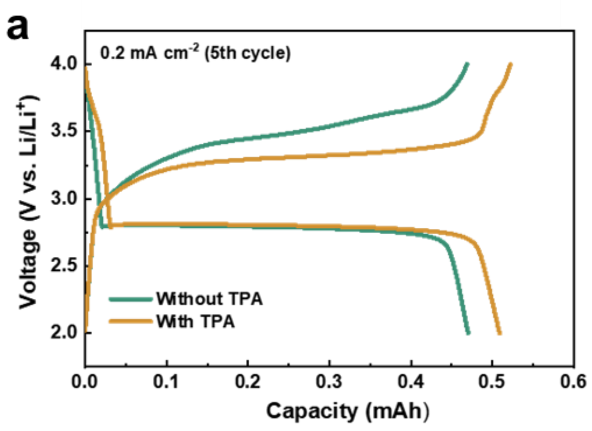

Figure S17. Charge and discharge curves of Li||KB cells containing SO_2-saturated electrolyte with and without TPA at current densities of a) 0.2 mA cm^{-2} and b) 5.0 mA cm^{-2} (refer to the rate capability comparison in Figure 3e).

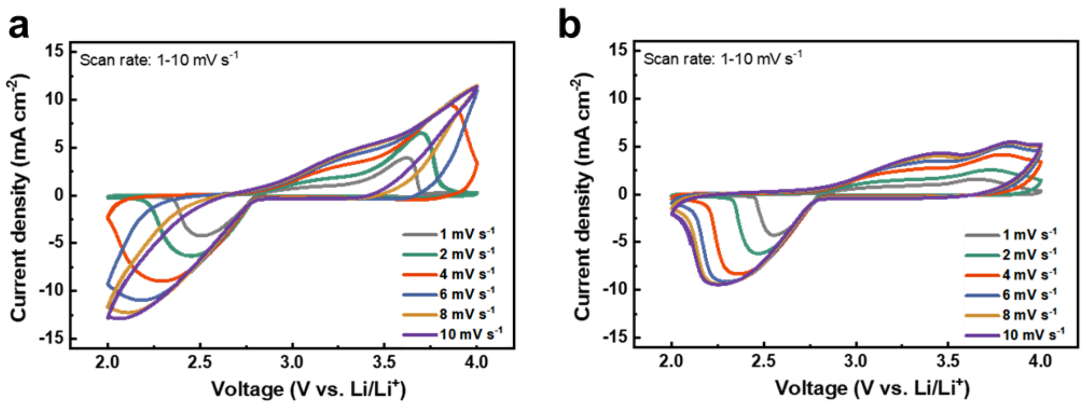

Figure S18. CV curves at scan rates from 1 mV s^{-1} to 10 mV s^{-1} for cells containing SO_2-saturated electrolyte a) with and b) without TPA.

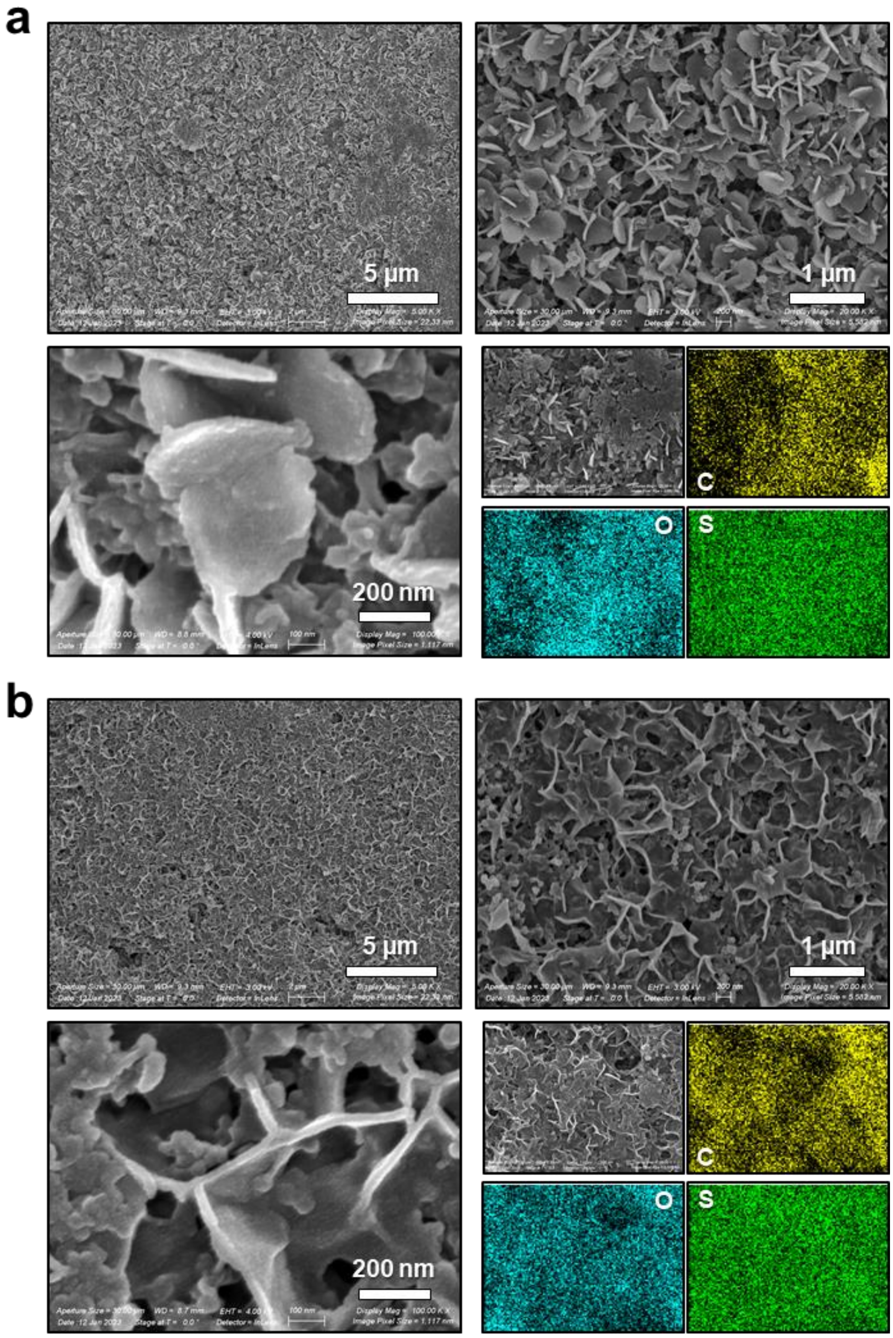

Figure S19. Top-view SEM and elemental mapping images of the KB cathode surface after the second discharge for cells containing SO_2-saturated electrolyte a) with and b) without TPA.

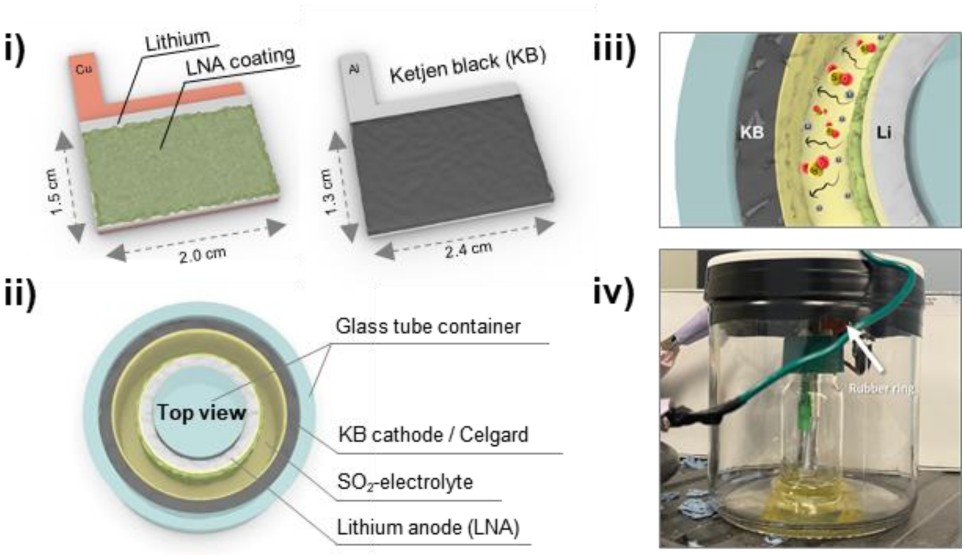

Figure S20. Schematic illustrations of i) two electrodes and ii) top-view of the demonstrated cell configuration. iii) An illustration depicting the anticipated transport mechanism of reactants between the LNA-Li and KB electrodes, along with iv) a digital image of a proof of concept bobbin-type cell.

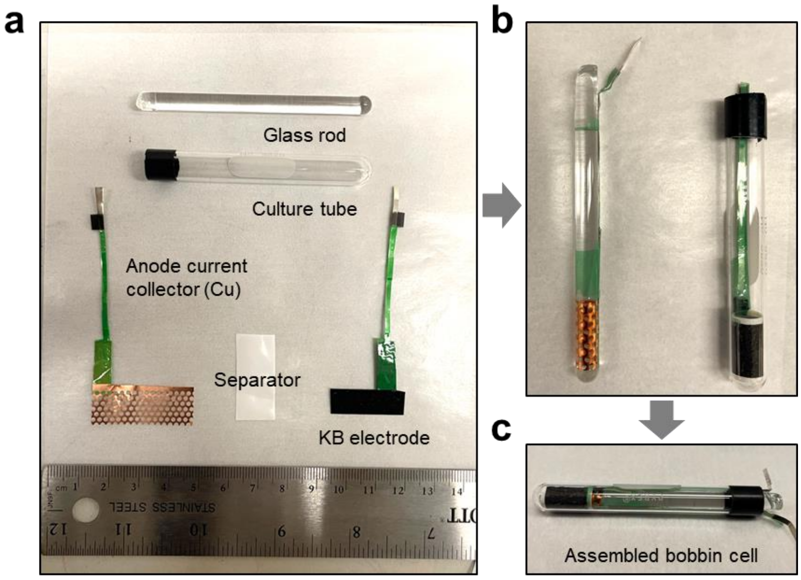

Figure S21. Digital photographs of bobbin cell components. a) Cross-sectional images displaying each listed component (glass rod, culture tube, anode, separator, and cathode). b) The anode and cathode rolled and assembled with a glass rod and culture tube, and c) the fully assembled bobbin cell within a single culture tube. Electrolyte is added later, and the cell is sealed with a rubber ring.

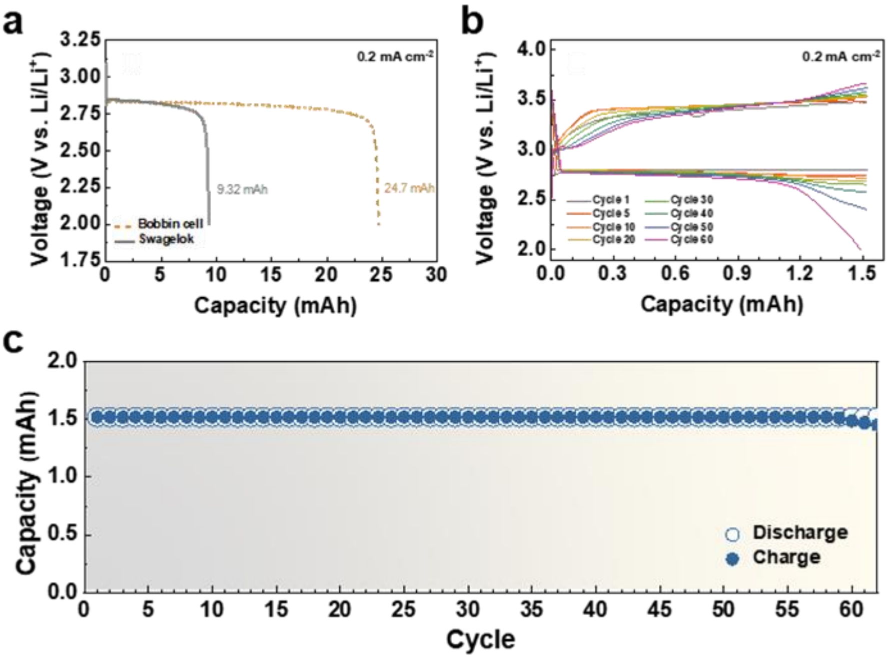

Figure S22. a) Discharge curves of Swagelok and bobbin cells containing LNA-Li and a SO_2-containing electrolyte with TPA at a current density of 0.2 mA cm^{-2}. b) Cycling characteristics with a fixed capacity of 1.52 mAh at a current density of 1.0 mA cm^{-2}, and c) capacity changes with respect to the number of cycles. To ensure that the specific capacity for KB is consistent (1.6 mAh cm^{-2}) when compared to the configuration demonstrated in Figure 4b for the bobbin cell, the limited capacity for cycling was set accordingly.

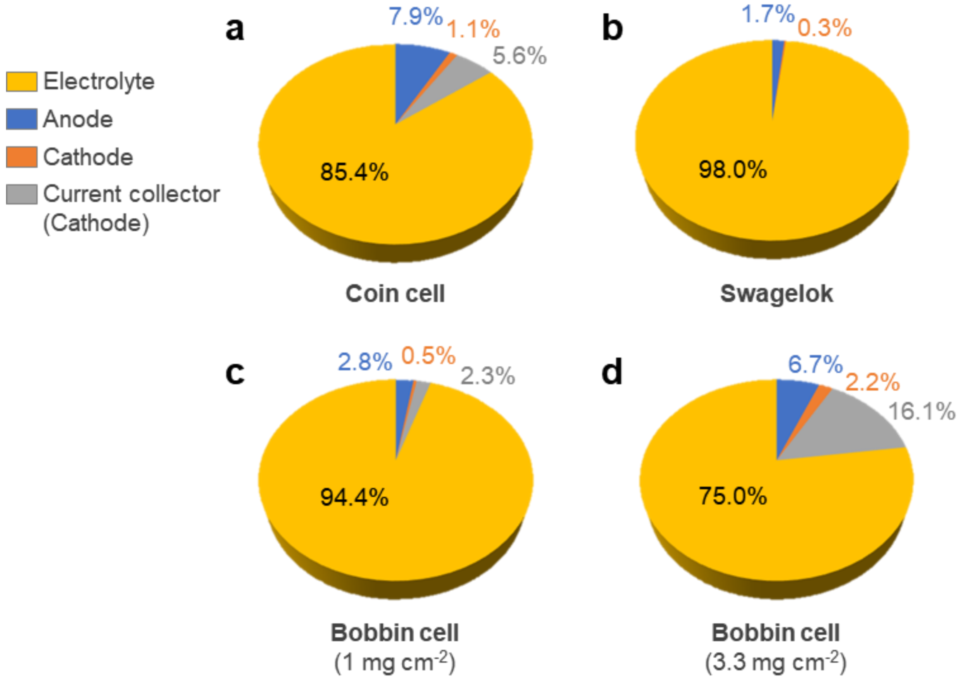

Figure S23. Mass distribution of battery cell components for various cell formats. a) Coin cell, b) Swagelok, and bobbin-cells with cathode loadings of c) 1 mg cm^{-2} and d) 3.3 mg cm^{-2}.

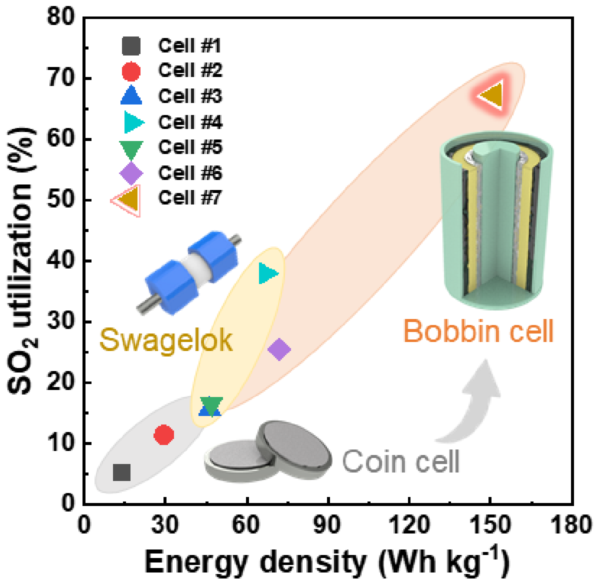

Figure S24. SO_2 utilization plotted against energy density, calculated based on experimentally measured capacities and the actual weight of the electrodes used for various cell configurations (see Table S3 for details).

			Weight (g)	Volume (ml)	Density (g/ml)	Li concentration (M)
1 M LiTFSI	EC/DMC	Bare	10.03	7.5	1.34	1
		SO_2-bubbled	14.94	11.0	1.36	0.68
	DME	Bare	8.54	8.0	1.07	1
		SO_2-bubbled	13.30	12.5	1.06	0.64

Table S1. Changes in weight, volume, and density after SO_2 dissolution in two different electrolytes.

Cell format	Cell configuration	KB weight (mg cm^{-2})	Electrolyte weight (g)	Theoretical capacity (mAh)	Experimental capacity (mAh)	Utilization (%)	Specific capacity (mAh g_{KB}^{-1})
Coin cells	Li	0.9	0.076	8.11	0.43	5.30	476.3
	Li (GF/F)				0.93	11.47	983.1
	LNA-Li				0.87	10.73	1023.3
Swagelok	LNA-Li (with TPA)	3.3	0.354	37.76	9.32	24.68	2972.9
Bobbin type cells	Li	1.0	0.354	37.76	6.26	16.58	3260.0
	Li	1.9			9.64	25.53	2509.0
	Li	3.3			24.24	64.19	2585.5
	Li (with TPA)				24.70	65.41	2638.9
	LNA-Li (with TPA)				25.40	67.27	2713.7

Table S2. Comparison of SO_2 utilization and specific capacity for different cell configurations, including cell format with or without the LNA-lithium protective layer and TPA.

Cell format		Coin cells (Cell #1, #2)		Swagelok (Cell #3, #4)		Bobbin type cells (Cell #5, #6, #7)		
**Cell configuration**	Anode	Li (100 µm)	Li (100 µm, GF/F)	Ref. 15 Li (100 µm)	LNA-Li (200 µm)	Li (100 µm)	Li (100 µm)	LNA-Li (200 µm)
	Cathode	KB (0.9 mg cm^{-2})	KB (0.9 mg cm^{-2})	KB (0.8 mg cm^{-2})	KB (3.3 mg cm^{-2})	KB (1.0 mg cm^{-2})	KB (1.9 mg cm^{-2})	KB (3.3 mg cm^{-2})
	Current collector	Al foil	Al foil	Carbon paper	Stainless steel gauze	Al foil	Al foil	Stainless steel gauze
**Anode**	mg	7.03	7.03	7.03	10.1	10.6	10.6	31.8
**Cathode**	mg	1.02	1.02	1.01	3.48	1.92	3.69	10.42
**Current collector**	mg	4.97	4.97	-	23.06	8.44	8.44	75.75
**Electrolyte**	g	0.076	0.076	0.398	0.354		0.354	
**Total weight**	g	0.090	0.090	0.406	0.391	0.375	0.377	0.472
**Total capacity**	mAh	0.433	0.929	6.680	9.320	6.260	9.635	25.396
**Energy density (2.81 V vs. Li/Li^+)**	**Wh/kg**	**13.67**	**29.33**	**46.18**	**66.80**	**46.91**	**71.87**	**151.20**
**E/C ratio**	**g/Ah**	**175.52**	**81.81**	**59.64**	**37.98**	**56.55**	**36.74**	**13.94**

Table S3. Energy densities and E/C ratios determined from the measured capacities and the actual weights of the electrodes.

Supporting information note 1. Calculation of the theoretical specific capacity of electrolyte saturated with SO_2.

$$\text{Anode: } Li(s) \rightarrow Li^{+} + e^{-}$$

$$\text{Cathode: } 2SO_2(g) + 2e^{-} \rightarrow S_2O_4{}^{2-}$$

$$\text{Total: } 2Li(s) + 2\,SO_2(g) \rightarrow Li_2S_2O_4(s)$$

1) Charge of 1 M electrons: $(6.023 \times 10^{23})\left(\frac{1}{\text{mol}}\right) \times (1.602 \times 10^{-19})(\text{C})$

$$= 96488\left(\frac{\text{C}}{\text{mol}}\right) = 26.80\ Ah/mol$$

2) Weight of SO_2

$$(32.07 \times 1)(\text{g/mol}) + (16.00 \times 2)(\text{g/mol}) = 64.07\ \text{g/mol}$$

3) Capacity (Q) of 1g of SO_2

Since 2 moles of SO_2 react with 2 moles of lithium, the capacity (Q) when 1g of SO_2 reacts is calculated as follows.

$$Q = \frac{\left(26.80\frac{Ah}{mol}\right) \times 2}{\left(64.07\frac{g}{mol}\right) \times 2} \cdot \frac{1000\ mAh}{Ah} = 418.29\ \text{mAh}$$

4) Theoretical specific capacity of electrolyte

SO_2 concentration value obtained by titration when using a SO_2-saturated solution (25.5 wt%):

$$418.29\frac{mAh}{g_{SO2}} \times \frac{25.5}{100} = 106.66\ \text{mAh}/g_{electrolyte}$$

Supporting information note 2. Projected energy density of an optimized single-layer Li–SO_2 bobbin cell using 1M LiTFSI EC/DMC (1:1 v/v) electrolyte saturated with SO_2.

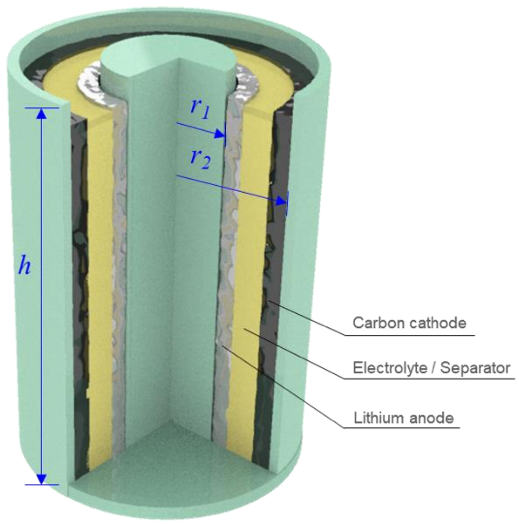

r_1 : radius of center part
r_2 : inner radius of cell container
h : height of cell container
C_{SO2} : expected capacity per mass of electrolyte

ρ_{el} : density of SO_2-saturated electrolyte (1.36 g/ml)
ρ_{Cu} : density of copper (8.96 g/ml)
ρ_{Al} : density of aluminum (2.7 g/ml)
ρ_{se} : density of separator (0.895 g/ml)
ε_{se} : porosity of separator (0.4)
ρ_{LNA} : density of LNA coating
ε_{LNA} : porosity of LNA coating

1) SO_2-saturated electrolyte

Assumption 1. The thickness of the anode and cathode is negligible compared to (r_2 - r_1).

Volume of electrolyte (V_{el}) = Area × height = $\pi \cdot h \cdot (r_2^2 - r_1^2)$

Weight of electrolyte (W_{el}) = $\rho_{el} \cdot \pi \cdot h \cdot (r_2^2 - r_1^2) = \rho_{el} \cdot V_{el} = 1.36 \cdot V_{el}$

Assumption 2. SO_2 utilization = 67.3 % → $C_{SO2} = 106.66 \cdot 0.673 = 71.78\ \mathrm{mAh}/g_{electrolyte}$

Electrolyte capacity (C_{el}) = $C_{SO2} \cdot \rho_{el} \cdot V_{el} = 97.62 \cdot V_{el}$

2) Lithium anode

Assumption 3. N/P ratio = 2 → Anode capacity = $2 \cdot C_{el} = 2 \cdot C_{SO2} \cdot \rho_{el} \cdot V_{el}$

Weight of anode (W_{an}) = $\frac{Anode\ capacity}{3860\ mAh/g_{Li}} = \frac{2 \cdot C_{SO2} \cdot \rho_{el}}{3860\ mAh/g_{Li}} \cdot V_{el} = 0.05 \cdot V_{el}$

3) KB cathode

Assumption 4. Limited KB electrode = 6500 mAh g_{KB}^{-1}, based on references [1], [15]

Assumption 5. Cathode is composed of 90% of KB and 10% of PTFE binder in weight ratio

Weight of cathode (W_{ca}) = $\frac{1}{0.9} \cdot (weight\ of\ KB) = \frac{1}{0.9} \cdot \frac{C_{SO2} \cdot \rho_{el}}{6500\ \mathrm{mAh}\ g_{KB}^{-1}} \cdot V_{el} = 0.02 \cdot V_{el}$

4) Redox-inactive components: current collectors, separator, LNA coating

Assumption 6. Thickness of current collectors and separator (t_1) = 15 µm

Assumption 7. Thickness of LNA coating (t_2) = 70 µm, ρ_{LNA}= 3.18 g/ml, ε_{LNA} = 0.9

Weight of anode current collector ($W_{cc.Cu}$) = $\rho_{Cu} \cdot \mathrm{t}_1 \cdot 2\pi \cdot r_1 \cdot \mathrm{h}$

$$= 8.96 \cdot (15 \cdot \frac{cm}{10000\ \mu m}) \cdot 2\pi \cdot r_1 \cdot \mathrm{h}$$

Weight of cathode current collector ($W_{cc.Al}$) = $\rho_{Al} \cdot \mathrm{t}_1 \cdot 2\pi \cdot r_2 \cdot \mathrm{h}$

$$= 2.7 \cdot (15 \cdot \frac{cm}{10000\ \mu m}) \cdot 2\pi \cdot r_2 \cdot \mathrm{h}$$

Weight of separator (W_{se}) = $(1 - \varepsilon_{se}) \cdot \rho_{se} \cdot \mathrm{t}_1 \cdot 2\pi \cdot r_2 \cdot \mathrm{h}$

$$= 0.6 \cdot 0.895 \cdot (15 \cdot \frac{cm}{10000\ \mu m}) \cdot 2\pi \cdot r_2 \cdot \mathrm{h}$$

Weight of LNA coating (W_{LNA}) = $(1 - \varepsilon_{LNA}) \cdot \rho_{LNA} \cdot \mathrm{t}_2 \cdot 2\pi \cdot r_1 \cdot \mathrm{h}$

$$= 0.1 \cdot 3.18 \cdot (70 \cdot \frac{cm}{10000\ \mu m}) \cdot 2\pi \cdot r_1 \cdot \mathrm{h}$$

Weight of all other components (W_{other}) = $W_{cc.Cu} + W_{cc.Al} + W_{se} + W_{LNA}$

$$= \pi \cdot \mathrm{h} \cdot (0.03 r_1 + 0.01 r_2)$$

5) Projected energy density

Assumption 8. Nominal voltage of SO_2 redox reaction = 2.81 V *vs.* Li/Li^+

Total energy (Wh) = $C_{el} \cdot 2.81 = (97.62 \cdot \frac{Ah}{1000\ mAh} \cdot 2.81) \cdot V_{el} = 0.27 \cdot V_{el}$

Assumption 9. $W_{other} = \delta \cdot (\text{total weight}), 0 < \delta < 1$

Total weight (kg) = $W_{el} + W_{an} + W_{ca} + W_{other}$

$$= \frac{1}{(1-\delta)} \cdot (W_{el} + W_{an} + W_{ca}) = \frac{1}{(1-\delta)} \cdot (1.36 + 0.05 + 0.02) \cdot \frac{kg}{1000\ g} \cdot V_{el}$$

$$= \frac{1}{(1-\delta)} \cdot (1.43 \cdot 10^{-3}) \cdot V_{el}$$

Projected energy density = $\frac{Total\ energy\ (Wh)}{Total\ weight\ (kg)} = \frac{(1-\delta) \cdot 0.27 \cdot V_{el}}{(1.43 \cdot 10^{-3}) V_{el}} = (1 - \delta) \cdot 188.81\ Wh/kg$

6) Projected energy density: when $\boldsymbol{\delta = 0.03}$

$$\therefore \text{Projected energy density} = (1 - \delta) \cdot (188.81)(\frac{Wh}{kg}) = 183.15\ Wh/kg$$

Supporting information note 3. Proposed cell design of a sealed rechargeable Li−SO_2 battery.

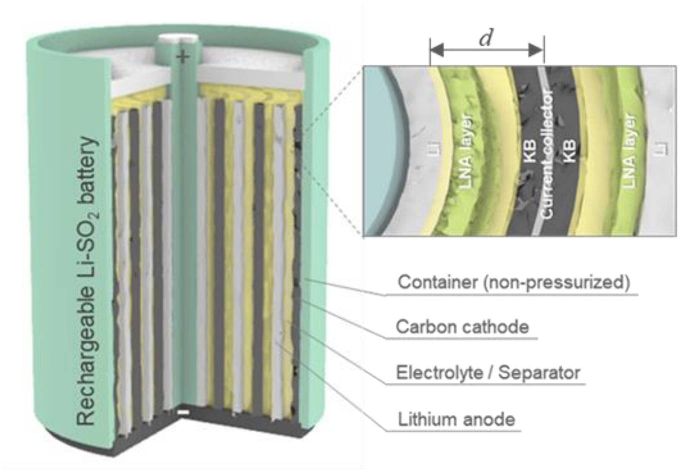

This cell consists of multiple layers of alternating anodes and cathodes arranged in an interdigitated configuration and packaged in a cylindrical cell. The lithium electrode is coated with an LNA protective layer, and the carbon cathode is deposited onto a porous current collector. The SO_2-containing electrolyte includes TPA additives. Considering SO_2 utilization, the distance (d) between anode and cathode is determined based on the targeted electrolyte volume thus energy density. This sealed Li–SO_2 battery is engineered to attain high energy density and prolonged cycling stability without requiring an external gas inlet port, making it a promising low-cost rechargeable battery free of any transition metals.
